# Antibodies to a conformational epitope on gp41 neutralize HIV-1 by destabilizing the Env spike

**DOI:** 10.1038/ncomms9167

**Published:** 2015-09-25

**Authors:** Jeong Hyun Lee, Daniel P. Leaman, Arthur S. Kim, Alba Torrents de la Peña, Kwinten Sliepen, Anila Yasmeen, Ronald Derking, Alejandra Ramos, Steven W. de Taeye, Gabriel Ozorowski, Florian Klein, Dennis R. Burton, Michel C. Nussenzweig, Pascal Poignard, John P. Moore, Per Johan Klasse, Rogier W. Sanders, Michael B. Zwick, Ian A. Wilson, Andrew B. Ward

**Affiliations:** 1 Department of Integrative Structural and Computational Biology, The Scripps Research Institute, La Jolla, California 92037, USA; 2 International AIDS Vaccine Initiative Neutralizing Antibody Center and the Collaboration for AIDS Vaccine Discovery (CAVD), The Scripps Research Institute, La Jolla California 92037, USA; 3 Center for HIV/AIDS Vaccine Immunology and Immunogen Discovery, The Scripps Research Institute, La Jolla, California 92037, USA; 4 Department of Immunology and Microbial Science, The Scripps Research Institute, La Jolla, California 92037, USA; 5 Department of Medicinal Microbiology, Academic Medical Center, 1105 AZ Amsterdam, The Netherlands; 6 Weill Medical College of Cornell University, New York, New York 10065, USA; 7 Laboratory of Molecular Immunology, The Rockefeller University, New York, New York 10065, USA; 8 Ragon Institute of MGH, MIT and Harvard, Boston, Massachusetts 02114, USA; 9 Howard Hughes Medical Institute, The Rockefeller University, New York, New York 10065, USA; 10 Skaggs Institute for Chemical Biology, The Scripps Research Institute, La Jolla, California 92037, USA

## Abstract

The recent identification of three broadly neutralizing antibodies (bnAbs) against gp120–gp41 interface epitopes has expanded the targetable surface on the HIV-1 envelope glycoprotein (Env) trimer. By using biochemical, biophysical and computational methods, we map the previously unknown trimer epitopes of two related antibodies, 3BC315 and 3BC176. A cryo-EM reconstruction of a soluble Env trimer bound to 3BC315 Fab at 9.3 Å resolution reveals that the antibody binds between two gp41 protomers, and neutralizes the virus by accelerating trimer decay. In contrast, bnAb 35O22 binding to a partially overlapping quaternary epitope at the gp120–gp41 interface does not induce decay. A conserved gp41-proximal glycan at N88 was also shown to play a role in the binding kinetics of 3BC176 and 3BC315. Finally, our data suggest that the dynamic structure of the Env trimer influences exposure of bnAb epitopes.

## Abstract (web summary)

The envelope glycoprotein (Env) trimer is the only antigenic target for broadly neutralizing antibodies on the surface of the HIV-1 virus. Here the authors show that two related monoclonal antibodies bind between gp41 protomers and neutralize HIV-1 by accelerating Env trimer decay.

## Graphical abstract

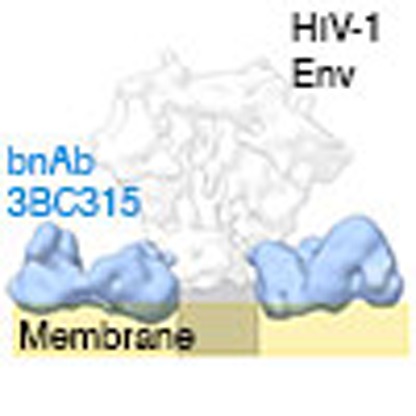

The envelope glycoprotein (Env) of human immunodeficiency virus type-1 (HIV-1) is required to recognize and infect host cells and is the only target on the surface of the virus for antibody-mediated neutralization. Env is a heavily glycosylated trimeric assembly of non-covalently associated gp120 and gp41 heterodimers, which arise from proteolytic cleavage of the gp160 polypeptide. Protein-shielding glycans and high Env sequence variability enable HIV-1 to effectively evade the human immune system and eventually lead to AIDS if untreated. Despite these obstacles, some HIV-1-infected patients develop antibodies over time that potently neutralize a wide range of circulating HIV-1 strains^1^,^2^,^3^,^4^,^5^. In the past few years, functional screening and B-cell sorting technologies have identified many such broadly neutralizing antibodies (bnAbs)^4^,^6^,^7^,^8^. Electron microscopy (EM) and X-ray crystallographic studies of these bnAbs in complex with Env subunits and trimers have resulted in a wealth of information regarding a wide range of complex epitopes on Env. The recent demonstration that structure-based immunogens for respiratory syncytial virus (RSV) can elicit protective antibodies in immunized animals^9^ has further galvanized ongoing efforts to induce HIV-1 neutralizing antibodies. Thus, a comprehensive understanding of the sites of vulnerability on HIV-1 Env and how antibodies develop to recognize these sites has become increasingly valuable for structure-based immunogen design^10^,^11^.

The majority of known bnAbs target one of four epitope clusters on the surface of Env that are often composed of both peptide and glycan components. These sites include the receptor, or CD4, binding site (CD4bs)^3^,^4^, the quaternary epitope surrounding the N160 glycan at the apex of the trimer^1^,^6^,^12^,^13^, the high-mannose patch on the outer domain of gp120 that includes the N332 glycan at the base of variable loop 3 (V3)^1^,^14^,^15^,^16^ and the membrane-proximal external region (MPER) of gp41 (ref. 2). In addition, three new bnAbs have been classified: PGT151 (refs 17, 18), 35O22 (ref. 19) and 8ANC195 (ref. 20). These antibodies all target conserved sites that incorporate peptide and glycan segments from both gp120 and gp41. Their identification fills in some of the few remaining gaps in bnAb coverage of the Env trimer surface. The PGT151 and 35O22 epitopes, in particular, are highly dependent on the quaternary structure of the closed, pre-fusion form of Env^13^,^17^,^18^,^19^.

Two moderately broad and potent, clonally related monoclonal antibodies isolated from a single donor, 3BC176 and 3BC315, recognize a glycan-independent epitope that was proposed to be located in the vicinity of the V3 loop and CD4-induced (CD4i) site^5^. Potency and breadth of these antibodies were tested on a panel of 39 viruses representing most clades. Thirteen of the viruses in the panel were isolates resistant to 3BNC117 and 3BNC55, highly potent CD4bs antibodies that were isolated from the same donor. Out of the 39 viruses tested, 3BC176 (median IC_50_=1.69 μg ml^−1^) and 3BC315 (median IC_50_=10.00 μg ml^−1^) neutralized 25 diverse isolates, and were complementary to 3BNC117 and 3BNC55, as they neutralized 10 of 13 strains that were resistant to these CD4bs antibodies^5^.

In this study, we characterize the elusive 3BC176/3BC315 epitopes by structural methods using soluble BG505 SOSIP.664 gp140 trimers, which display multiple bnAb epitopes^21^. Structural analyses of the 3BC315 and 3BC176 fragment antigen binding (Fab) by X-ray crystallography, and in complexes with BG505 SOSIP.664 by single-particle cryo-electron microscopy (cryo-EM), reveal that the 3BC315 and 3BC176 epitopes are very similar and situated at the interface between two gp41 subunits. The antibodies bind near the base of the trimer close to, but distinct from, the epitope of the 35O22 bnAb at the gp120–gp41 interface. Like 35O22, the 3BC176/3BC315 epitopes do not require the MPER to bind, and they interact with the soluble trimer primarily via heavy chain (HC) interactions. Our in-depth biophysical analyses of the 3BC315–Env interactions illustrate how such a complex, glycan-shielded epitope in gp41 may be recognized. Furthermore, we show that the binding of 3BC176/3BC315 to native, HIV-1 virion-associated Env causes the trimers to become destabilized and disintegrate suggesting a possible mechanism of virus neutralization.

## Results

### 3BC315 is a gp41–gp41 interprotomer-binding antibody

The 3BC176 and 3BC315 epitopes have remained undefined using commonly used epitope mapping methods, such as competition enzyme-linked immunosorbent assays (ELISAs) and mutagenesis assays. On the other hand, structural studies can provide a direct picture of the epitopes, which is particularly helpful in characterizing quaternary epitopes that involve more than one protomer of the trimer and are, therefore, difficult to delineate using point mutation binding assays. Several gp120–gp41 interface-binding antibodies, such as PGT151, 35O22 and 8ANC195 (refs 17, 18, 19, 20), have been described indicating that new epitopes are still being discovered. On generating reference-free two-dimensional (2D) class averages of BG505 SOSIP in complex with 3BC315 and 3BC176, it was clear that these antibodies bound much more similarly to 35O22 than a typical CD4i antibody such as 17b (Supplementary Fig. 1a). In addition, binding of soluble CD4 (sCD4) to the trimer neither improve 3BC315 nor 3BC176 binding to SOSIP.664_293T_ trimer by ELISA (Supplementary Fig. 1b), unlike 17b (ref. 21).

**Figure 1:**
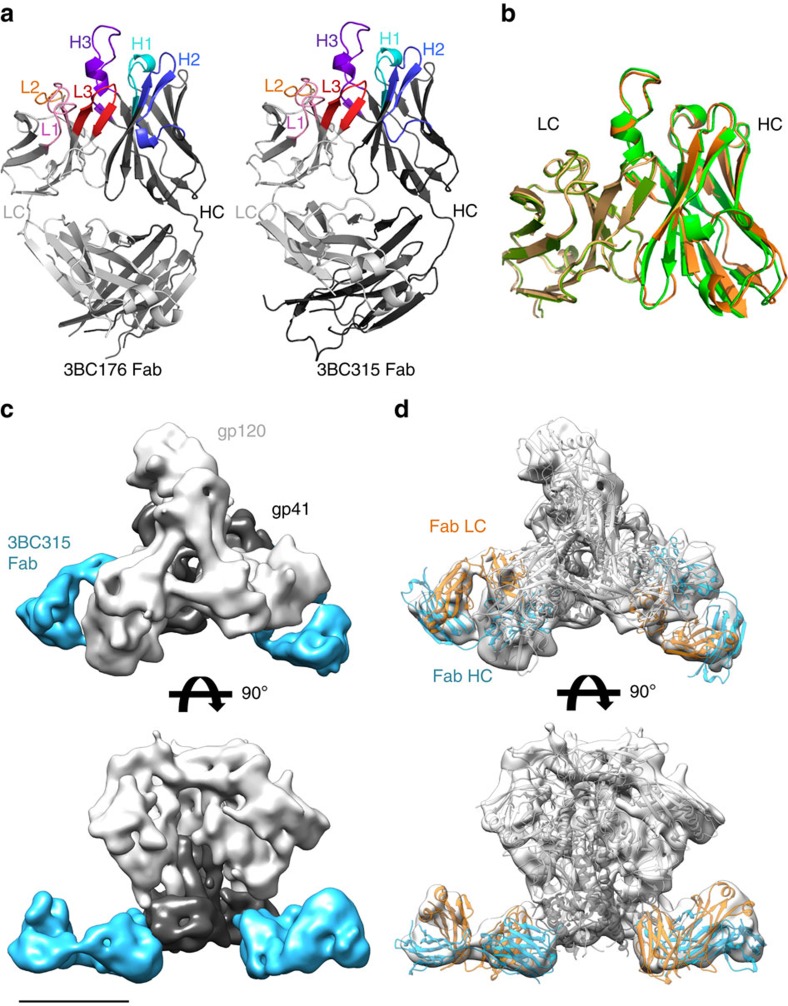
Structural analysis of 3BC176/3BC315 paratopes, and their epitopes on the BG505 SOSIP.664_293F_ trimer. (**a**) X-ray structures of 3BC176 and 3BC315 are shown in cartoon rendering. The Fab heavy chain (HC) and light chain (LC) are shown in dark and light grey, respectively. The CDR loops are coloured and labelled. (**b**) Cα superimposition of the 3BC176 and 3BC315 Fab variable regions. 3BC315 is shown in orange (HC) and yellow (LC) and 3BC176 in green. (**c**) Single-particle cryo-EM reconstruction of 3BC315 Fab in complex with the BG505 SOSIP.664_293F_ trimer. Only two Fabs are bound per trimer. The map has been segmented in Chimera into gp120 (light grey), gp41 (dark grey) and Fab (blue) using coordinates from PDB ID: 4TVP for the SOSIP Env trimer and the Fab structure as determined here. The scale bar corresponds to 50 Å. (**d**) Three copies of gp140 and two copies of 3BC315 Fab structures were fit independently into the EM map (white surface). The gp120 and gp41 are shown as light and dark grey ribbons, respectively. Fab LC and HC are coloured in orange and blue. The small protruding bumps on the Env surface correspond to glycans.

To study the paratopes of 3BC315 and 3BC176, their corresponding Fabs were crystallized and their structures were determined to 1.95 and 1.89 Å resolution, respectively (Fig. 1a; Supplementary Fig. 2a,b and Supplementary Table 1). The complementarity-determining regions (CDRs) of the two Fabs are in very similar conformations with Cα root mean squared deviation of 0.58 Å (Fig. 1b) for the variable regions, suggesting that the two Fabs likely interact with Env in a similar manner. Most of the CDR loops adopt canonical CDR conformations, with the light chain (LC) CDRs (CDRL) 1 and 2 being similar to class 6 and class 1, respectively, and heavy chain CDRs (CDRH) 1 and 3 belonging to class 1 and class 3, respectively^22^,^23^,^24^. Only CDRL3 and CDRH3 did not correspond to any categorized class. Like many HIV-1 bnAbs, the 3BC315 and 3BC176 Fabs have long CDRH3s, both being 19 residues, in comparison to the average human CDRH3 length of ∼15 residues^25^.

**Figure 2:**
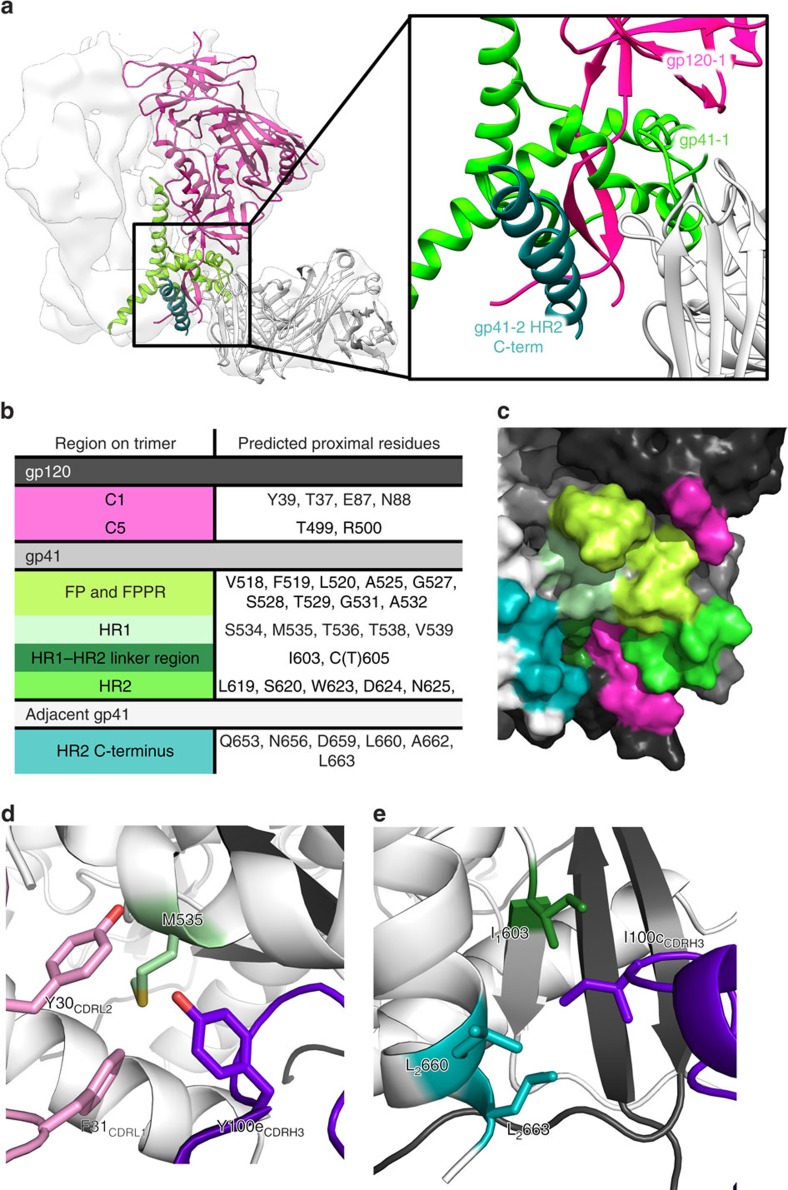
Identification of the 3BC315 epitope by cryo-EM. (**a**) On the basis of the docking of crystal structures into the EM reconstruction (white surface), 3BC315 Fab (white cartoon) interacts with gp41 (gp41-1, light green) and gp120 (gp120, pink) within a single gp140 protomer, as well as with the adjacent gp41 (gp41-2, dark green) of the neighbouring protomer. A close up view of this region is shown in the inset. (**b**) Following RosettaRelax, all side chains in the Env trimer within 5 Å of the antibody CDR loops are shown here. The gp41 residue-forming part of the SOS bond (C605) is also in close proximity. (**c**) Residues and regions of the trimer that are contacted or buried by the Fab (listed in (**b**)) are shown on gp120 and gp41 (represented in black and dark grey, respectively) of one protomer, as well as on the adjacent gp41 (white). The 3BC315 epitope is coloured by region as in (**b**) on the surface rendering of the trimer. (**d**) M535 in gp41 is surrounded by aromatic residues from CDRH3 (purple) and CDRL1 (pink). (**e**) I100c from CDRH3 inserts itself into a hydrophobic pocket in the gp41–gp41 interface. Possible interacting residues are labeled and the subscript distinguishes between residues from two gp41 protomers of the trimer.

Of the two Fabs, 3BC315 bound the BG505 SOSIP.664 trimer produced in 293F cells with higher occupancy than 3BC176 (Supplementary Fig. 1a). Hence, the 3BC315 Fab: BG505 SOSIP.664_293F_ complex was chosen for cryo-EM structure determination (Fig. 1c; Supplementary Fig. 1c–e). Following incubation with tenfold molar excess of Fab both before and after purification of the complex by size-exclusion chromatography (SEC), the maximum stoichiometry observed was 2 Fabs bound per BG505 trimer. Particles belonging to this population were reconstructed into a 3D map (Fig. 1c) at 9.3 Å resolution, as determined by a gold standard Fourier shell correlation at a cutoff of 0.143 (Supplementary Fig. 1e). Characteristic features that are readily identifiable in the reconstruction at this resolution included the α-1 helix of gp120, the gp41 heptad repeat (HR) 1 helix at the centre of the trimer, and the HR2 helix at the C-terminus, as observed in the published high-resolution structures of BG505 SOSIP.664 (refs 26, 27, 28). The trimer structure with the most complete model of gp41 was used for docking purposes, and the position of the aforementioned regions in the reconstruction superimposes with the same regions in this X-ray trimer structure (Supplementary Fig. 1f)^28^ (PDB ID: 4TVP) after rigid-body docking of the trimer (Fig. 1d). The 3BC315 Fab crystal structure was then docked into the EM reconstruction to identify elements involved in its interaction with gp41 (Fig. 1d). The majority of possible contacts map to gp41 HR2, with potential interactions between gp120 and the C-terminus of the adjacent gp41 protomer (Fig. 2a). While some minor clashes were apparent between 3BC315 and HR2, the paratope of the Fab has high shape complementarity to the pre-fusion conformation of gp41, and is not compatible with gp41 in the six-helical bundle post-fusion conformation^29^.

In an attempt to resolve potential molecular interactions between the trimer and the antibody, we performed rigid-body docking followed by RosettaRelax^30^, using the EM density as a spatial constraint. We analysed potential contacts to the trimer within a liberal contact radius of 5 Å around Fab CDR residues (Fig. 2b,c). Some significant hydrophobic interactions were observed, such as the hydrophobic region formed by aromatic residues of CDRL1 and CDRH3 (Fig. 2d), and I100c in CDRH3, which inserts into a hydrophobic gp41–gp41 interprotomer region (Fig. 2e). Next, we looked for sequence conservation patterns in gp41 between isolates that were neutralized and those that were not, in the original neutralization panel^5^ (Supplementary Fig. 3a). With a few exceptions, only residue 619 stood out, where more than half of the isolates not neutralized by 3BC315 or 3BC176 had an aromatic residue (Tyr or Phe) at this position (Supplementary Fig. 3b).

### Mapping of the gp41 interprotomer epitope

**Figure 3:**
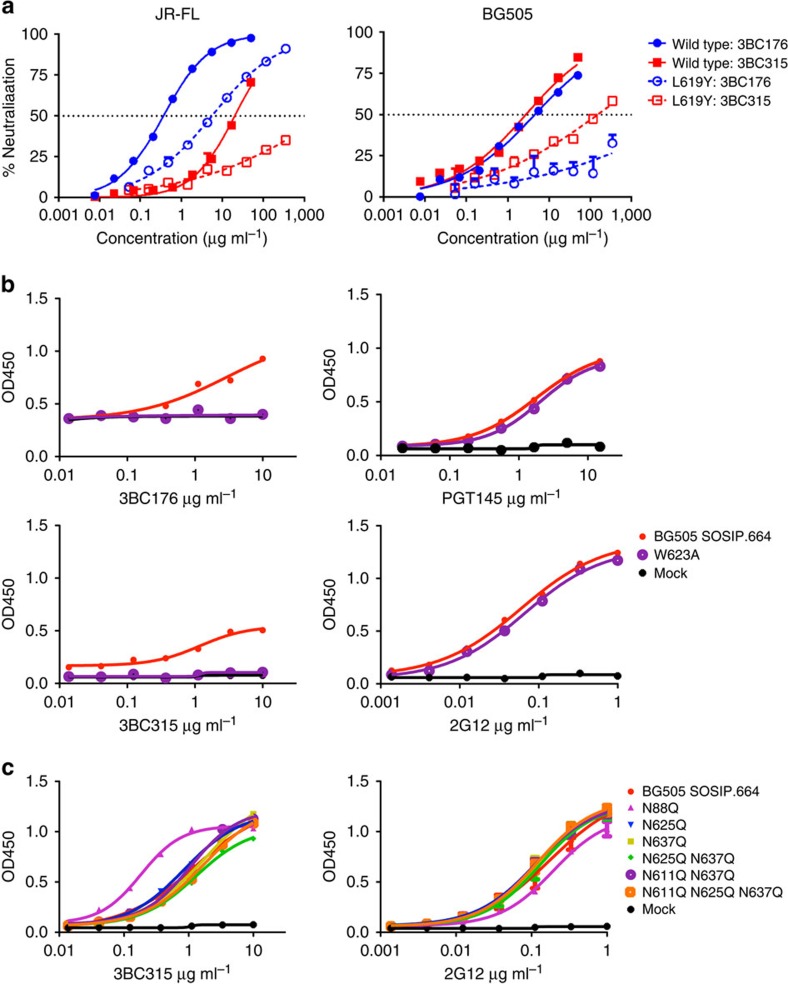
The 3BC315 epitope involves gp41 HR2 residues at positions 619 and 623, and is partially shielded by a gp120 glycan. (**a**) Neutralization of wild type and L619Y mutant JR-FL (left) and BG505 (right) pseudoviruses by 3BC176 and 3BC315 IgG, measured in a TZM-bl neutralization assay. Error bars shown indicate s.d. (**b**) Binding of 3BC176 and 3BC315 to wild type and W623A BG505 SOSIP.664_293T_ measured by ELISA. This mutation in the heart of the epitope clearly reduces 3BC176 and 3BC315 binding to the SOSIP trimer. Binding of the trimer-specific antibody PGT145 confirms that the W623A mutant does not adversely affect the quaternary structure of the trimer. (**c**) Binding of 3BC315 Fab measured by ELISA to BG505 SOSIP.664_293T_ variants with 3BC315 epitope proximal glycan knockouts. 2G12 was used as a control as its binding is not dependent on any of the knocked out glycans. Error bars shown indicate s.e.m.

We endeavoured to define the epitope in further detail and scanned gp41 regions via point mutagenesis and using TZM-bl neutralization assays on JR-FL/JR2 or BG505 background pseudoviruses for 3BC176 and 3BC315. On the basis of the observed binding site of 3BC315 in the EM reconstruction, JR-FL pseudoviruses with single substitutions in regions of gp41 including the disulphide loop, HR2 and MPER, as well as C5 of gp120 were tested for sensitivity to 3BC176 and 3BC315 IgG (Supplementary Table 2a,b). Neutralization sensitivity was most affected for 3BC176, where IC_50_ values against disulphide loop and C5 mutants in JR-FL or JR2 were surprisingly decreased (that is, potency increased) by fourfold to tenfold relative to wild type, and none of these mutations conferred resistance to 3BC176. In contrast, mutation L619Y in HR2 increased the 3BC176 IC_50_ against JR-FL and BG505 15-fold and >78-fold, respectively. The same mutation increased the 3BC315 IC_50_ against JR-FL and BG505 >18-fold and 55-fold, respectively (Fig. 3a). Other mutations in HR2 resulted in large IC_50_ increases, in accord with their position in the EM model. Substitutions at positions W623 and E648 in HR2 of JR-FL resulted in >40-fold increase in IC_50_ of 3BC176 (Supplementary Table 2a), and another point substitution N656A resulted in a 13-fold IC_50_ increase. E648 and N656 within the C-terminal half of HR2 are proximal to W623 from the N-terminal half of HR2 from an adjacent protomer (Fig. 2b,c) consistent with the inter-gp41 epitope visualized by cryo-EM. However, the same mutations had a smaller effect on 3BC315. While this may be due to the fact that 3BC315 does not neutralize JR2 and JR-FL as well as 3BC176, it also attests to the complex nature of the quaternary epitope, where amino acids from different protomers play a role in defining the conformational epitope. In addition, while the 3BC176/3BC315 epitope is mainly confined to gp41, 3BC176/3BC315 did not bind various gp41 linear peptides and gp41 constructs (Supplementary Table 3). In this assay, 3BC176/3BC315 bound only ADA gp140 I559P, which has the gp41-pre-fusion stabilizing I559P mutation similar to SOSIP. Selective binding to ADA gp140 I559P, but not ADA gp140, indicates that 3BC176/3BC315 only recognize the pre-fusion conformation, as the I559P substitution prevents gp41 from going into its helical post-fusion form. This preference for binding pre-fusion gp41 is discussed in further detail below.

Alanine mutagenesis scans of the HR2 region were also carried out on BG505 SOSIP.664_293T_ trimers to determine if 3BC315 bound the stabilized SOSIP trimers differently from cell-surface Env (Fig. 3b; Supplementary Fig. 4a), and because mutating W623 in the BG505 virus rendered the virus non-infectious. 2G12 was used as a control, along with the trimer apex-binding antibody PGT145. The majority of the point mutations had only a small effect on the binding of 3BC315 as well as the control antibodies. Nevertheless, in accord with the neutralization assay data for 3BC176, the W623A substitution abolished binding by 3BC315 (Fig. 3b). In the recent high-resolution X-ray structure of the BG505 SOSIP.664 trimer, W623, along with W628 and W631, form a “tryptophan clasp” surrounding M530 that is proposed to stabilize the pre-fusion conformation^28^. In our model, CDRH3 is in close proximity to W623 (Supplementary Fig. 5a). Overall, the main molecular recognition between the CDR loops and the residues that most strongly affect antibody binding and neutralization seemed to be based mainly on van der Waals interactions rather than specific polar interactions. Lastly, while the binding location at the base of the trimer suggests that the Fab may interact with MPER, adding back the MPER to the SOSIP_293T_ trimer resulted only in a modest improvement in 3BC315 binding by ELISA (Supplementary Fig. 4b).

The 3BC176 Fab structure was docked into the 3BC315 complex structure via superimposition to explain differences in some of their neutralization specificities. We mapped the residues that are different in the CDR loops between the two antibodies that could potentially make direct contacts within our Rosetta model. These residues were Y33H, N52D, N53V and F56I in the HC (3BC176 amino acid is listed after the residue number) (Supplementary Fig. 5b), and S27aN and N93S in the LC (Supplementary Fig. 5c). The likely contacts of the HC residues are within gp41 residues 620–625, where the 620, 624 and 625 side chains point towards the CDRH loops (Supplementary Fig. 5b). Although interaction with these residues does not seem to be the primary determinant for neutralization, residue 53 in the HC is an asparagine in 3BC315, while it is a valine in 3BC176, indicating that sequence variability of gp41 residues in with region may affect antibody potency.

The only likely contacts of the two LC residues were in the fusion peptide (FP) in a region that corresponds to the six N-terminal gp41 residues that are absent in the model derived from the crystal structure. The pocket formed by CDRL1, CDRL3 and CDRH3 is largely hydrophobic and could accommodate the hydrophobic FP (Supplementary Fig. 5d). Because S27a and N93 are both also hydrophilic in 3BC176, it is difficult to predict what role these residues have in FP recognition without a high-resolution atomic model. Overall, the differences in the mode of binding and epitope recognition that we observe by the two antibodies is small, and is difficult to explain, as is for groups of numerous other HIV-1 bnAbs that belong to the same lineage, for which we see variation in neutralization potency towards different viral isolates^1^,^4^,^17^,^31^.

### Dual nature of glycan dependent 3BC315 epitope recognition

HIV-1 antibodies with long CDR loops typically incorporate glycans as part of their epitope. Despite the close proximity of Fab CDRH3 to potential N-glycosylation sites (PNGS) N88 and N625, in the previous work by Klein *et al.*^5^, glycan array analysis did not show any glycan binding to 3BC315 or 3BC176. Consistent with these data, 3BC315 showed only a small difference in binding by ELISA when SOSIP.664_293T_ trimers were produced in the presence of kifunesine (Supplementary Fig. 6a). The gp41 glycan knockout mutations in both JR-FL/JR2 virus (Supplementary Table 2a) and BG505 SOSIP.664_293T_ (Fig. 3c; N611, N616/618, N625 and N637) subtly affected neutralization potency or binding of 3BC176 and 3BC315. The greatest change in neutralization potency and binding resulted from deletion of the N88 glycan in C1 of gp120 near the gp120–gp41 interface. The N88A substitution in JR-FL lead to ∼30-fold increased neutralization by 3BC176 and 3BC315, and a significant increase in 3BC315 binding to BG505 SOSIP.664_293T_ by ELISA (Fig. 3c; Supplementary Table 2a). The N88 glycan in the 35O22-bound crystal structure (PDB ID: 4TVP)^28^ would extensively clash with 3BC315 Fab if N88 remained fixed in the observed position (Supplementary Fig. 6b). Because N88 is conserved in nearly all viruses (NXT/S sequence 99% conserved in all HIV-1 sequences in the Los Alamos database), including those that were neutralized by 3BC176/3BC315 (ref. 5), it suggests that this glycan undergoes large movements and adopts a different conformation to accommodate 3BC315 binding.

When BG505 SOSIP.664 trimers produced in 293S cells were deglycosylated by endoglycosidase H (EndoH; Supplementary Fig. 6c), there was no significant difference in K_*d*_ values as measured by isothermal titration calorimetry (ITC; glycosylated: 185.7±33.8 nM; deglycosylated: 167.3±14.6 nM). However, 3BC315 Fab did bind the deglycosylated trimer with an increased stoichiometry of *N*=1.71, in comparison to *N*=1.17 for the fully glycosylated trimer (Table 1a; Supplementary Fig. 6d). The binding of 3BC315 Fab to glycosylated trimers also resulted in broadening of the thermal heat signature after the first few injections in comparison to that of the deglycosylated trimers (Supplementary Fig. 6e). When the same ITC experiments were carried out in SOSIP.664 trimers produced in 293F cells that produce complex glycans, the stoichiometry was further reduced (*N*=0.76), although the K_*d*_ was slightly improved (130.3±32.5 nM; Table 1a; Supplementary Fig. 6d). We propose that this effect may be due to a decreased on-rate resulting from steric blockade of the epitope by the surrounding glycans. Finally, when the last 14 residues of gp41 in the BG505 SOSIP.664 construct were truncated (BG505 SOSIP.650_293F_), 3BC315 Fab bound with ∼3-fold higher affinity by ITC (*N*=1.13, K_*d*_=43.1±18.3 nM; Table 1a; Supplementary Fig. 6d). Because the antibody must wedge in between two gp41 protomers to bind, we suspect that the deletion of the last 14 residues removes this steric barrier, and results in the improved affinity. Thus, the C-terminal helix appears to influence the 3BC315 epitope in the context of the trimer in much the same way as surrounding glycans can block/inhibit access by bnAb to their epitopes^16^,^32^,^33^,^34^.

We proceeded to test our hypothesis that glycans affect binding kinetics by surface plasmon resonance (SPR). 3BC315 IgG and Fab were both titrated against the BG505 SOSIP.664_293T_ trimer and the binding was modelled kinetically (Table 1b; Supplementary Fig. 7a,b). The Fab binding agrees well with the monovalent component of the modelled IgG binding. The on-rate constant was moderate and the off-rate constant extremely low, yielding a high affinity interaction (K_*d*_ <10 nM; Table 1b and Supplementary Fig. 7a,b). Both IgG and Fab gave low S_m_ values of 1.1, in agreement with the sub-stoichiometric occupancy observed by EM and ITC. In accord with the ITC data, the binding stoichiometry was the lowest for trimers for which glycans are more highly processed (293T/F, S_m_=1.1 and 1.3, respectively), with slight increases in 293S-produced trimers (S_m_=1.5) and even more when 293S trimers are deglycosylated (S_m_=1.9). As shown by the above modelling and ITC-binding signals (Supplementary Fig. 6d,e), the 3BC315 Fab-bound deglycosylated trimers with an on-rate constant, *k*_on_, that is ∼10-fold higher than for the natively glycosylated trimer (293T/F), and ∼6.5-fold higher than for the high-mannose glycan trimer (293S) (Table 1b). To our surprise, the increase in *k*_on_ was accompanied by an increase in the off-rate constant, *k*_off_. The binding to EndoH-treated 293S trimers, especially, showed a marked increase in the off-rate by at least tenfold; whereas the off-rate was too low to model accurately for the 293T/F trimers (Supplementary Fig. 7b).

### 3BC315 recognizes primarily gp41 within one gp140 protomer

**Figure 4:**
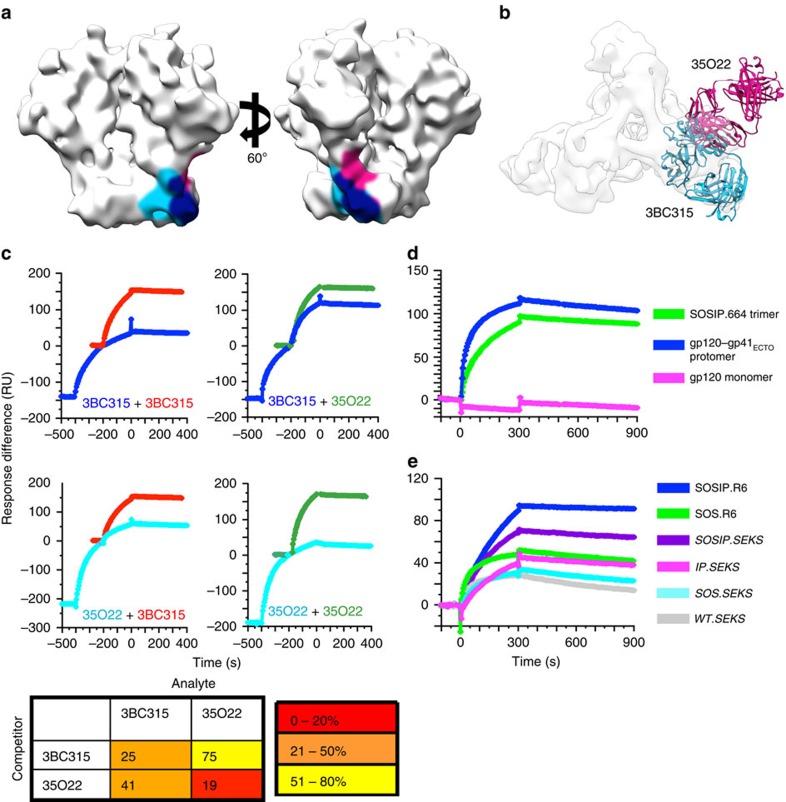
3BC315 binding to BG505 Env analysed by SPR. (**a**) The epitopes of 35O22 and 3BC315 are shaded on the surface of an unliganded SOSIP.664 cryo-EM structure (EMDB ID: 5782). The epitopes of 3BC315 (light blue) and 35O22 (pink) extensively overlap (navy). (**b**) 35O22 (pink) and 3BC315 (blue) bind the trimer from two different approach angles despite a large overlap in their epitopes. (**c**) SPR analysis of sequential antibody binding to BG505 SOSIP.664_293T_ trimer. The sequential-injection curve is colour-coded with the label of its first Ab. The single-injection curve for the second Ab in the sequential injection is superimposed on its corresponding curve in the second injection in a different colour. The curves are displayed in the same order as shown in the matrix to the right. The matrix diagram gives the binding of the second Ab in a sequential injection relative to its binding when injected alone (% of plateau colour-coded as shown in key at the bottom). The values are the means of two replicate experiments with s.e.m. values <5% of the means. (**d**) The 3BC315 IgG reactivity with BG505 SOSIP.664_293T_ trimer, gp120–gp41_ECTO-293T_ protomer and gp120_293T_ monomer is shown. All gp140 constructs shown here have the SOS and IP mutations. (**e**) The 3BC315 IgG reactivity with equivalent amounts of 293T-produced BG505 SOSIP trimers and cleavage-modified variants. Variants containing the R6 motif are fully cleaved, whereas those with *SEKS* are uncleaved.

The overall peptide epitope footprint of 3BC315 overlaps that of 35O22 by approximately 341 Å^2^ (∼35% of the entire 3BC315 epitope and ∼52% of 35O22 epitope; Fig. 4a), although the two antibodies bind from two very different angles of approach (Fig. 4b). Binding assays by SPR were performed to compare the degree to which 3BC315 and 35O22 compete with each other, and quaternary epitope preference of 3BC315 relative to quaternary-specific gp120–gp41 interface antibodies, PGT151 and 35O22 (refs 17, 18, 19). To assess any binding competition between 3BC315 and 35O22 by SPR^35^, the two antibodies were injected sequentially in a single cycle, the second antibody at the end of the association phase of the first (Fig. 4c). At the starting point of the second injection, the corresponding response curve for the second antibody when injected alone was superimposed on the combined curve. If the second curve for the sequential injection is lower than that for the same antibody injected alone, this indicates that the total number of binding sites for the second antibody is reduced, which can be expressed as the relative plateau (%). Concentrations of the two antibodies were set to give similar self-competition, resulting in 20–25% relative binding. 3BC315 or 35O22 inhibited the binding of the other to varying degrees; pre-bound 35O22 reduced the subsequent binding of 3BC315 to 41%, and pre-bound 3BC315 reduced binding of 35O22 to 75%. Therefore, while the two binding sites partially overlap and result in some inhibition of second antibody binding, incomplete binding occupancy from the first injection leaves some sites on the trimer open for the subsequent association step. The weaker ability of 3BC315 to block 35O22 binding is likely due to the low occupancy and on-rate of 3BC315 as seen by ITC and SPR, thereby leaving numerous sites unoccupied after the first injection.

3BC315 binding to BG505 SOSIP.664_293T_ trimer, gp120–gp41_ECTO-293T_ protomer, and gp120_293T_ monomer showed a consistent trend. Binding was stronger to the protomer than to the trimer and negligible with gp120 (Fig. 4d). This phenomenon is similar to the inhibitory effect by glycans, in which removal of some regions/features of the native trimer, such as certain highly conserved glycans, can increase antibody affinity by increasing accessibility to the epitope^16^,^32^,^33^,^34^. However, this binding preference is distinct from other gp120–gp41 interface antibodies, PGT151 (ref. 36) and 35O22 (ref. 19), both of which bind better to the trimer than to the protomer by the same assay. Finally, binding of 3BC315 to six variants of BG505 Env produced in 293T cells was compared (Fig. 4e). The constructs differ in their ability to be cleaved by furin (R6: hyper-scissile; *SEKS*: not cleaved), gp120–gp41 stability (SOS: gp120–gp41 disulphide present), and pre-fusion conformation preference (IP: adopts pre-fusion conformation), and have been previously described^36^,^37^. 3BC315 binding ranked as follows: it was strongest to SOSIP.R6; second strongest to *SOSIP.SEKS*, showing the importance of cleavage for optimal antigenicity. Binding to SOS.R6 and *IP.SEKS* was further reduced, the on-rate being lowest for the latter. The binding was lowest to *SOS.SEKS* and *WT.SEKS.* In conclusion, the SOSIP modifications and cleavage all contribute to the recapitulation of the binding site, where cleavage and the IP modification are particularly important.

### 3BC176/3BC315 accelerates Env trimer decay

**Figure 5:**
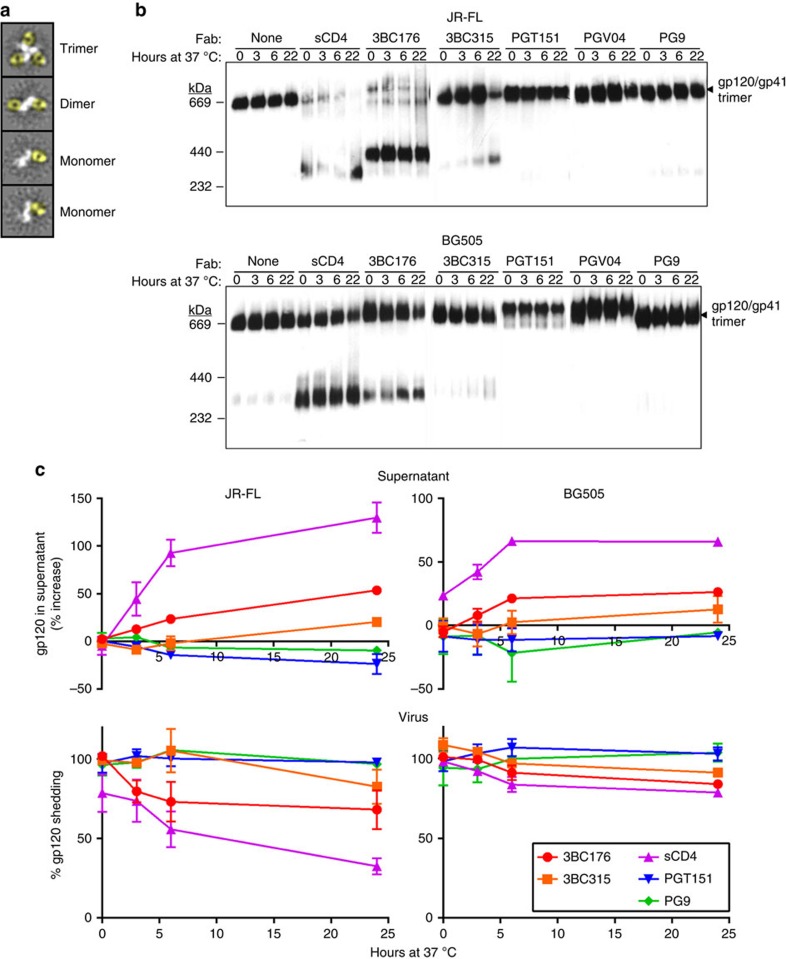
3BC176 and 3BC315 accelerate viral spike decay. (**a**) Representative 2D class averages seen for dissociated dimers and monomers. Compared with the trimeric Env portion of the protein complex in the top-most class average shown, the dimers and monomers have smaller mass indicating trimer dissociation. Fabs have been coloured in yellow for clarity. The white density corresponding to the SOSIP trimer becomes smaller as the trimer dissociates. Class averages here are generated using the 18-h time point from the JR-FL SOSIP.664: 3BC176 Fab complex. (**b**) JR-FL and BG505 virions were pre-incubated at 37 °C alone or with 20 μg ml^−1^ sCD4, 3BC176, 3BC315, PGT151, PGV04 or PG9 Fabs for various time points. Env was then solubilized by 1% DDM, separated using BN-PAGE, and visualized by western blot. (**c**) JR-FL and BG505 virions were incubated at 37 °C alone or with 20 μg ml^−1^ sCD4, 3BC176, 3BC315, PGT151 or PG9 Fabs for noted time points. Virions were then pelleted and gp120 shed into the cell culture supernatant (upper panels) or associated with virus (lower panels) was detected using ELISA. The % of increase in gp120 (upper panels) and the % of gp120 remaining with the virus (lower panels) are relative to the untreated control. The scales are not the same in all figures. In both measurements, gp120 shedding is observed when the virus is treated with sCD4 (control), 3BC176 or 3BC315, but not when treated with PG9 or PGT151. Error bars indicate s.d.

In our negative-stain EM analysis of both 3BC176 and 3BC315 binding to SOSIP.664_293F_ trimers, we observed that a fraction of the trimer population had dissociated into monomers, despite the fact that BG505 SOSIP.664 is known to be extremely stable^13^. Negative-stain EM analysis of 3BC176/3BC315 Fab in complex with either BG505 SOSIP.664 or JR-FL SOSIP.664 trimers at multiple time points after trimer-Fab mixing indicated that, over an 18-h period, the trimer was dissociated into Fab-bound gp140 dimers or monomers when incubated with 3BC176 or 3BC315 (Fig. 5a, Table 2). Conversely the positive control, PGV04, did not cause trimer dissociation (Table 2). We also sought to investigate trimer destabilization in a more biological context by testing the effect of antibody on the stability of viral surface Env. First, Fabs of various antibodies, including 3BC317 and 3BC315 were co-incubated with virus (JR-FL and BG505) at 37 °C for various lengths of time, and the Env–Fab complexes were then solubilized from the membrane using 1% dodecyl β-D-maltoside (DDM) to allow separation of the extracted complexes using blue native-PAGE (BN-PAGE) (Fig. 5b). The control ligand, sCD4 caused dissociation of JR-FL and BG505 Env trimers as expected, while little or no trimer dissociation was observed for control antibodies, PGT151, PGV04 or PG9. It should be noted that, in this assay, dissociation might be occurring during the gel run after detergent has been added, particularly with JR-FL, which is reportedly sensitive to CD4-induced shedding^38^. With JR-FL, 3BC176 also destabilized the trimer rapidly, inducing complete dissociation within three hours (Fig. 5b). 3BC315 also caused significant dissociation of the Env trimer, but the process was not as rapid as with 3BC176 (Fig. 5b). BG505 was more resistant to 3BC176 and 3BC315 destabilization than JR-FL, but both antibodies caused dissociation of the BG505 Env trimer over time (Fig. 5b,c).

Certain types of Abs, such as those that bind the MPER, have previously been shown to accelerate gp120 shedding^38^. We utilized a similar assay in an attempt to detect the degree of gp120 loss from the surface of the virus by measuring both the amount of gp120 released into the supernatant and that remaining on the virus by ELISA (Fig. 5c). The results were generally in accord with the BN-PAGE data and confirm that 3BC176 and 3BC315 accelerated the shedding of gp120 into cell culture supernatant. In conclusion, these data suggest that, relative to other non-MPER bnAbs, 3BC176 and 3BC315 both disrupt trimerization and induce gp120 shedding.

We also studied the ability of 3BC176 and 3BC315 to destabilize functional Env using a pre-incubation neutralization assay, in which virus is pre-incubated with IgG up to 22 h rather than the standard one hour before adding to target cells. If an antibody induces irreversible decay of the Env trimer over time, then it will result in the IC_50_ of that antibody decreasing the longer that it is incubated with virus. We found that 3BC176 and 3BC315 neutralization of JR-FL improved (IC_50_ decreased) roughly 8.7- and 7-fold, respectively, while neutralization of BG505 increased 5.8- and 4-fold over the 22 h incubation time (Table 3). Overall, membrane-associated trimers in the neutralization assays appeared to disintegrate slower than detergent-extracted trimers. In each case, the rate of IC_50_ decrease for 3BC176 and 3BC315 was greater than the positive control ligand sCD4 (5- and 2.6-fold), while little trimer decay over time was seen in the presence of the fusion inhibitor T20, and negative control antibodies D5 and 2G12. Additional Abs, PGT145, 2G12 and 35O22 were also tested for comparison. Most importantly, 35O22, which has the largest epitope overlap with 3BC176/3BC315 out of the antibodies tested, did not seem to cause trimer decay. Altogether, these results indicate that both 3BC176 and 3BC315 have a significant destabilizing effect on the functional Env trimer and this property likely contributes to their mechanism of neutralization.

## Discussion

Three recently characterized bnAbs have been shown to bind to antigenic sites outside of the four well-established sites of vulnerability and incorporate regions from both gp120 and gp41 subunits in their epitopes^17^,^18^,^19^,^20^. Here we describe yet another epitope on the Env trimer for the previously described 3BC176/3BC315 antibodies whose epitopes had not been defined molecularly^5^. While alanine-scanning mutagenesis provided some clues about the location of the epitope, our cryo-EM structure clearly revealed its location at the gp41–gp41 inter-subunit interface on the pre-fusion conformation of the Env trimer. On the basis of our modelling of this complex, the 3BC315 recognizes Env mainly via hydrophobic interactions and shape complementarity in a quaternary-dependent manner.

**Figure 6:**
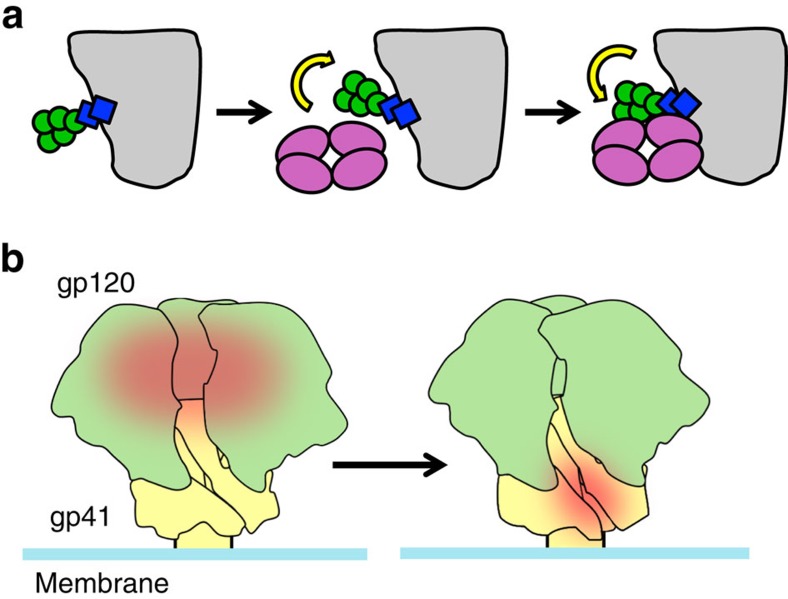
An overview of how 3BC315 interacts with the Env trimer. (**a**) The glycans near the 3BC315 epitope (e.g. N88) in the unliganded trimer reconfigure, enabling the antibody to bind to its epitope (left, centre). An increased on-rate is observed when these glycans are removed. In the glycosylated state, these same glycans lock the antibody in position, as indicated by low off-rate relative to deglycosylated trimers. (**b**) Model of the interplay between distant epitopes on the Env trimer. When the CD4bs is readily accessible (red patch), perhaps in early Env populations, the 3BC315 epitope is occluded (left). Occlusion of the CD4bs, potentially as a mechanism to escape CD4bs antibodies, then leads to Env variants in which the 3BC315 epitope (red patch) on gp41 (right) becomes exposed, perhaps via glycan reconfiguration.

The 3BC176/3BC315 epitope at the base of the Env trimer partially overlaps with the 35O22 epitope, although each antibody assumes a different angle of approach (Fig. 4a,b). 35O22 is highly dependent on a glycan at the gp120–gp41 interface, namely at N88. 3BC176/3BC315, on the other hand, have slower on- and off-rates in the presence of the glycan. While it is not uncommon for early precursors of bnAbs to avoid glycans^16^,^32^,^34^, 3BC176/3BC315 differs from all bnAbs outside the CD4bs^26^ and MPER, in that the mature antibody does not require glycans to bind. The high conservation of the N88 glycan may also explain the relatively low potency of 3BC176/3BC315 (ref. 5), as it restricts access to the epitope. Intriguingly our SPR data suggest that, once bound, the glycan(s) surrounding the 3BC315 epitope may act like a clasp and lock the antibody in place, markedly affecting the off-rate. Thus, although an energetically unfavourable shift in the glycan position must occur for the antibody to bind, the glycan, now positioned on top of the antibody, may prevent the Fab from dissociating from the trimer (Fig. 6a).

In contrast to other quaternary antibodies^17^,^18^,^19^,^20^, we observed that 3BC176/3BC315 negatively impacts Env trimer stability. Virion-associated Env dissociates into monomers on binding to 3BC176/3BC315, while the same phenomenon was not as pronounced with soluble SOSIP.664_293F_, which lacks the MPER. Interestingly, deletion of the gp41 region just upstream from the MPER, corresponding to the C-terminal residues of HR2 (∼650–664), improves binding by 3BC176/3BC315. This region was also recently shown to exhibit rapid exchange by HDXMS^39^, suggesting a dynamic topology, which may restrict access to this antigenic region of the trimer. While MPER-binding antibodies have been shown to induce the CD4i conformation^40^,^41^, or destabilize the Env trimer^38^,^42^, the ability of 3BC176/3BC315 to induce trimer dissociation is unique for quaternary-preferring antibodies described to date^13^,^18^,^19^,^20^,^43^,^44^.

While 3BC176/3BC315 are not highly potent antibodies, their apparent mechanism of neutralization is unlike any other non-MPER bnAbs. 3BC176/3BC315 complement the CD4bs bnAbs (3BNC117/3BNC55) isolated from the same patient by neutralizing viruses that are resistant to this set of CD4bs bnAbs^5^. It is worth noting that the CD4bs bnAbs contain heavy chain framework 3 insertions that are predicted to interact with or clash at the interprotomer interface proximal to the CD4bs^26^,^45^. Thus, binding of these bnAbs may require some ‘breathing’ of the trimer, consistent with phenomena recently observed by single molecule studies of virion-associated Env^44^. Sequence analyses of susceptible versus resistant Env strains reveal no obvious basis for the reciprocal neutralization profile observed by Klein *et al.*^5^ (for example, there are no mutations in the CD4bs that would obviously result in escape). Therefore, we propose that escape may be mediated by mutations that alter trimer dynamics, and therefore accessibility to the CD4bs. This altered behaviour may then result in increased exposure of the antigenic region defined by 3BC176/3BC315, thereby inducing an orthogonal bnAb lineage (Fig. 6b) over time. This intriguing possibility suggests that one antibody lineage may significantly impact the evolution of another. In fact, a longitudinal study of virus and antibody evolution in a chronically infected HIV-1 patient has shown that bnAbs targeting various sites develop in a series of ‘waves’^46^, wherein the initial wave of neutralizing antibodies targeted the V1/V2 trimer apex, followed by CD4bs antibodies, and a third wave against a yet to be determined site. Cooperation between B-cell lineages was recently suggested as a mechanism for bnAb development^47^, albeit by antibodies that recognize overlapping epitopes. Our 3BC176/3BC315 data provide the first structural insights into the development of different broadly neutralizing specificities within one patient. Therefore, as *bona fide* trimeric Env immunogens that recapitulate Env structure continue to be developed^21^, it will be critical to incorporate considerations of Env epitope accessibility and trimer dynamics, as well as to carefully track evolution of immune responses to understand the complex interplay between epitopes.

## Methods

### Protein expression and purification

BG505 SOSIP.664 trimers were recombinantly expressed in HEK293T, 293F or 293S (GnTI^−/−^) cells, and 2G12-affinity purified^13^,^21^,^27^,^48^. The JR-FL SOSIP.664 trimers were produced in 293T cells and PGT145-affinity purified^49^. The supernatant was flown over Sepharose resin cross-linked to 2G12 or PGT145 IgG, then washed with 20 mM Tris (pH 8), 500 mM NaCl and eluted with 3 M MgCl_2_. The eluted trimers were dialyzed into 20 mM Tris (pH 8), 500 mM NaCl. BG505 SOSIP.664 trimers were further purified through a HiLoad 16/600 Superdex 200 SEC column (GE Healthcare) for structural and biophysical studies.

3BC315 and 3BC176 were expressed in HEK293F cells as Fabs or IgGs, where the HC and LC genes were co-transfected at a ratio of 2:1. The IgGs were purified in a single step, through a 5-ml protein A column (GE Healthcare). Fabs were purified in three steps. First, the collected media was loaded onto a 5-ml Lambda Select column (GE Healthcare), followed by cation exchange chromatography using a Mono S column (GE Healthcare). Fractions corresponding to the proper HC–LC dimer paring were then SEC-purified through a Superdex 200 column (GE Healthcare).

### Deglycosylation of BG505 SOSIP trimers

BG505 SOSIP.664 trimers expressed in 293S cells were deglycosylated using EndoH, by incubating with 0.3 units of EndoH (New England Biolabs) per μg of purified trimer, for 3 h at room temperature (RT) in 0.1 mM sodium acetate (pH 6)^48^. The deglycosylated trimer was further purified over Superose 6 (GE Healthcare), and checked for reduction in trimer mass by BN-PAGE 4–16% bis-Tris gels run using the manufacturer’s protocol (Novex).

### Crystallization and X-ray structure determination

3BC176 and 3BC315 Fabs SEC-purified in 50 mM Tris (pH 7.4), 150 mM NaCl and concentrated to ∼8 mg ml^−1^ were screened for crystallization conditions using the CrystalMation robot (Rigaku) at the Joint Center for Structural Genomics ( www.jcsg.org). Initial hits were optimized, and diffraction quality crystals were obtained in the following conditions: 3BC176: 6 mg ml^−1^ protein, 0.1 M sodium citrate (pH 5.6), 20% 2-propanol and 20% PEG4000; 3BC315: 8.5 mg ml^−1^ protein, 0.1 M *N*-cyclohexyl-3-aminopropanesulfonic acid (CAPS) (pH 10.3), 0.25 M NaCl and 20% PEG8000. Glycerol, 25% was used as a cryo-protectant for 3BC315 Fab.

Data sets for 3BC176 and 3BC315 were collected at liquid nitrogen temperatures at APS 23ID-D (wavelength: 1.033 Å) and APS 4.2.2 (wavelength: 1.000 Å) beamlines, respectively. Data processing was performed using HKL2000 (ref. 50). Both Fab structures were solved by molecular replacement^51^ using Fab coordinates of PDB ID: 4DJV for 3BC315 and the Fab coordinates of the 3BC315 structure for 3BC176, then refined using PHENIX^51^ and COOT^52^. Ramachandran statistics for the 3BC315 structure: Ramachandran favored/Ramachandran allowed: 97.5%/2.5%. Ramachandran statistics for the 3BC176 structure: Ramachandran favored/Ramachandran allowed: 96.7%/3.03%. Other statistics are reported in Table 1.

### Pseudovirus production and neutralization assays

The Env complementation vector pSVIIIex-E7pA^−^_YU2_ was kindly provided by J. Sodroski (Harvard) and the envelope genes JR-FL, JR2 and BG505 were cloned using the *KpnI* and *XhoI* restriction sites^53^. Envelope gene mutations were performed using a QuikChange site-directed mutagenesis kit (Agilent) according to the manufacturer’s protocol and verified through Sanger sequencing. HIV-1 pseudotyped viruses were produced in HEK293T cells by a polyethylenimine (PEI) co-transfection of the Env plasmid (PEI 25 K, Sigma-Aldrich) and backbone plasmid pSG3ΔEnv at a mass ratio of 1:3.5. Virus containing supernatant was collected 48 h post transfection, 0.2 μm filtered to remove cell debris and stored at −80 °C until use.

Single round neutralization assays were performed using TZM-bl cells (CD4^+^CXCR4^+^CCR5^+^), which were seeded at 10,000 cells per well 24 h before assay. The virus and antibody mixture were incubated for an hour at 37 °C before being added to cells. Luciferase activity was determined 48 h post infection and data were analysed using Prism 5.0 software (GraphPad). Extended incubation neutralization assays were performed as described above but with extended incubation times for the virus and antibody mixture at 37 °C (ref. 42). Some mutations were tested in a JR2 background instead of JR-FL. JR2 is identical to JR-FL except for the substitutions S668N, T676S and K677N^53^, and the neutralization potency of 3BC176 and 3BC315 was very similar against both JR-FL and JR2.

### gp120 shedding assay

Virus samples were incubated alone or with 20 μg ml^−1^ sCD4 or Fabs for various time points at 37 °C. Subsequently, virus was pelleted by centrifugation at 22,000 *g* for 45 min. gp120 content in both the cell culture supernatant and associated with the virus pellet was analysed using a gp120 capture ELISA. Briefly, samples were solubilized using 1% Empigen, gp120 was captured on anti-gp120 antibody D7324 (Aalto), and was then detected using purified IgG from pooled HIV-1 seropositive donors (HIVIG). The relative amount of gp120 in both the supernatant and associated with virus was normalized to virus samples incubated at 37 °C for the same time points without ligand.

### BN-PAGE binding and dissociation analysis

Virus samples were pre-incubated with Fab fragments of antibodies for the noted time periods before preparation for BN-PAGE. BN-PAGE analysis was performed using the NativePAGE bis-Tris gel system (Invitrogen), according to the manufacturer’s instructions. Briefly, samples were solubilized with 1% DDM in the supplied sample buffer and run on a 3–12% gradient bis-Tris NativePAGE gel at 150 V at 4 °C for 3 h. Proteins in the gel were transferred to a PVDF membrane; membranes were blocked in 5% (w/v) non-fat dry milk dissolved in PBS, 0.05% Tween-20, and blotted overnight at 4 °C using a cocktail of antibodies to gp120 (2 μg ml^−1^ each of b12, 2G12 and 447-52D) and to gp41 (1 μg ml^−1^ each of 2F5, 4E10 and Z13e1) combined. Membranes were washed, probed for 30 min at RT with an horseradish peroxidase (HRP)-conjugated goat anti-human Fc antibody (Jackson) and peroxidase activity was assayed using SuperSignal West Pico Chemiluminescence (Pierce).

### Ni-NTA ELISA

Pure HEK293T cell supernatants containing BG505 SOSIP.664, or PGT145 affinity-purified BG505 SOSIP.664 and BG505 SOSIP.MPER (0.5 μg ml^−1^), all with a C-terminal 8 histidine-tag were immobilized on Ni-NTA HisSorb 96-well plates (Qiagen) (100 μl per well) for 2 h at RT. After three washing steps with Tris-buffered saline (TBS), antibodies were serially diluted in 2% skim milk and added for 2 h. Subsequently, HRP labelled goat anti-human Abs (Jackson ImmunoResearch) were added for 1 h in TBS, 2% skimmed milk, followed by five washes with TBS, 0.05% Tween-20. Colorimetric detection was performed using a solution containing 1% 3,3′,5,5′-tetramethylbenzidine (Sigma-Aldrich), 0.01% H_2_O_2_, 100 mM sodium acetate and 100 mM citric acid. ELISA curves were fitted using Prism 5.01 (GraphPad).

### D7324-tag ELISA for gp120 binding and alanine scanning

Pure HEK293T supernatants containing D7324-tagged BG505 SOSIP.664 trimers and alanine scan mutants, or 2G12 affinity then SEC-purified BG505 SOSIP.664 trimers (0.5 μg ml^−1^) were coated on D7324-coated half-well ELISA plates (Greiner; 50 μl per well) for 2 h at RT. Subsequent ELISA assay steps were performed as described above for the Ni-NTA ELISA. In some cases, sCD4 (10 μg ml^−1^) was added during the incubation with a test antibody.

### Electron microscopy

The 3BC176, 3BC315 Fab or PGV04 Fab: SOSIP.664_293F_ complexes were generated by adding sixfold molar excess of the Fab to the trimer at RT for various time points. For negative-stain EM, the sample was diluted to ∼0.01 mg ml^−1^ immediately before applying to glow-discharged carbon coated 400-mesh Cu grids; 3 μl of the sample was applied for ∼2 s, followed by blotting and staining with 3 μl of 2% (w/v) uranyl formate for 30 s. Complexes were imaged on an FEI Tecnai T12 electron microscope operating at 120 kV at 52,000 × magnification, using a Tietz Temcam F-416 4 × 4 k camera, or on an FEI Talos electron microscope operating at 200 kV at × 73,000 magnification, using a 4 × 4 k Ceta camera. All data were collected via the Leginon interface^54^. Particle picking was carried out using Dogpicker^55^, and reference-free 2D class averages were generated using iterative multivariate statistical analysis/multireference alignments (MSA/MRA)^56^.

For cryo-EM, 3BC315 Fab was added to BG505 SOSIP.664_293F_ in tenfold molar excess, and incubated at 4 °C for 30 min, then purified over a Superose 6 column (GE Healthcare) and concentrated to ∼1.5 mg ml^−1^. DDM was added to the sample just before freezing such that the final DDM concentration in the solution was 0.018% (w/v). Grids were frozen manually at 4 °C, using 2/2 C-Flat holey grids. Data were collected using an FEI Tecnai F20 electron microscope operating at 200 kV, equipped with a Gatan K2 direct electron detector camera. The exposure images were collected in counting mode at × 29,000 magnification, at a pixel size of 1.21 Å at the specimen plane, receiving a total accumulated exposure dose of ∼27 e^−^/Å^2^. In the processed dataset, only frames 5–25 were aligned due to large alignment shifts in the first few frames. Particles were automatically selected, boxed out, and sorted by reference-free 2D class averaging as described above. CTF estimation was performed using CTFFind3 (ref. 57). The 2D-sorted particles were further sorted by two rounds of 3D classification using particles binned by 2, and using an unliganded trimer structure (EMD-5782)^26^ low-pass filtered to 60 Å as an initial model in RELION^58^. Particles from the two convergent classes that looked like Fabs complexed to an Env trimer were used to generate a final unbinned reconstruction. The final refinement was performed without symmetry using the RELION auto-refine function, with internal CTF correction^58^,^59^. The resolution of the model generated from 20,252 particles at a box size of 224 × 224 pixels (1.21 Å per pixel) was determined to be 9.3 Å by a gold standard Fourier shell correlation curve using a correlation cutoff of 0.143, following the application of a soft edge mask^60^. A B-factor of −600 Å^2^ was applied to obtain the final sharpened reconstruction.

### Structural analysis of cryo-EM reconstruction

The Segment Map function in UCSF Chimera was used to segment the refined map into gp120, gp41 and Fabs as seen in Fig. 1c^61^. Three copies of the gp140 protomer (PDB ID: 4TVP)^28^ and two copies of 3BC315 Fab were fit independently using the Fit function. Buried surface area for the epitopes of 3BC315 and 35O22 were calculated using the buriedArea function in Chimera, using the 4TVP trimer structure. 3BC315 Fab and the 4TVP trimer were docked into the BG505 SOSIP.664: 3BC315 Fab EM map to calculate the buried surface area. This resulted in solvent-excluded total buried surface areas of 986 and 657 Å^2^ for 3BC315 and 35O22, respectively. Glycans were excluded from the analysis. Figures were generated using Chimera^61^ and PyMOL^62^.

### Model refinement in Rosetta and analysis

The coordinates of the gp140 and Fab components that were fit in Chimera were saved as a single PDB file relative to the EM density and further refined by RosettaRelax^30^. Fifty models were generated. Because all Rosetta energy scores were highly clustered, the model resulting in the lowest energy after subtracting out the EM density score was used for analysis. Potential contacts were determined by selecting all residues in the trimer within a 5 Å radius around the Fab CDR loops in PyMOL. Only the contact residues that were identified from both interacting protomers are listed.

### Isothermal titration calorimetry

Samples were analysed on an Auto-iTC200 instrument (GE Healthcare). BG505 SOSIP.664 or SOSIP.650 trimers expressed in 293F or 293S cells were deposited in the cell at a concentration of 4.0–5.0 μM, and binding was titrated with the 3BC315 Fab in the syringe at ∼20 × the molar concentration of the trimer. Data analysis was carried out using the iTC200 software (GE Healthcare) using a single-site binding model.

### Surface plasmon resonance

Antibody binding was analysed by SPR on a Biacore 3000 instrument at 25 °C. Trimers were immobilized by antibody, either via a D7324-epitope or a 8 histidine-tag^36^. The D7324 antibody (Aalto Bio Reagents) was amide-coupled to a CM5 sensor chip to 9,000 resonance units (RU). After deactivation, D7324-tagged HEK293T expressed BG505 SOSIP.664 trimer, its SOSIP- and SEKS-modified variants, or gp120 gp41_ECTO_ protomer was captured to R_L_ values of 500 RU; gp120 monomer was similarly captured to give R_L_ values of 425RU, that is, 15% lower to achieve equal molar amounts. In all experiments, HBS-EP (10 mM HEPES (pH 7.4), 150 mM NaCl, 3 mM EDTA and 0.002% P20 surfactant) was used as running buffer (GE Healthcare). The flow rate for analyte injections was set to the maximum, 50 μl min^−1^, to minimize mass-transport limitation. Association was monitored for 5 min and dissociation for 10 min. After each cycle, the capture-antibody surface was regenerated by an injection of 10 mM glycine (pH 2) for 120 s at a flow rate of 30 μl min^−1^. In all these comparisons of different forms of D7324-tagged BG505 Env, 3BC315 was injected at 500 nM. The signal from a control channel, with capture antibody but no Env, was subtracted. For kinetic and competition analyses the more stable anti-His-capture was used. Anti-histidine antibody (GE Healthcare) was amide-coupled to the CM5 dextran surface to 15,000 RU. His-tagged BG505 SOSIP.664_293T/F/S_ Env was captured to R_L_ values of 500 RU in each cycle. 3BC315 (IgG or Fab) was injected at concentrations titrated down in twofold steps from 1 μM until no significant binding occurred. EndoH–deglycosylated 293S trimer was immobilized to a level proportionate to its lower mass (320 kDa), consistent with its mass reported previously^48^. The signals from both 0-analyte and control-channel injections were subtracted. In the kinetic experiments, IgG binding was fitted with a bivalent and Fab binding with a Langmuir model in BIAevaluation (GE Healthcare). For the sequential binding analyses, association and dissociation phases were monitored for 200 s each. 3BC315 and 35O22 were injected at 1,000 and 500 nM, respectively, either sequentially (at times 0 and 200 s in a cycle) or alone. Different concentrations were used so as to give similar self-inhibition, which resulted in residual 20–25% relative binding.

## Additional information

**Accession codes:** Atomic coordinates and structure factor files have been deposited in the Protein Data Band under accession codes 5CCK and 5AWN. The EM reconstruction has been deposited in the Electron Microscopy Data Bank under accession code EMD-3067.

**How to cite this article:** Lee, J. H. *et al.* Antibodies to a conformational epitope on gp41 neutralize HIV-1 by destabilizing the Env spike. *Nat. Commun.* 6:8167 doi: 10.1038/ncomms9167 (2015).

## Supplementary Material

Supplementary Information Supplementary Figures 1-7, Supplementary Tables 1-3 and Supplementary References

## Figures and Tables

**Table 1:** Kinetics of 3BC315 binding to BG505 SOSIP.664 trimers.

**(a)**	**SOSIP.664 293S**	**SOSIP.664 293S EndoH**	**SOSIP.664 293F**	**SOSIP.650 293F**
*N*	1.17±0.10	1.71±0.02	0.76±0.06	1.13±0.12
*K*_*d*_ (nM)	185.7±33.8	167.3±14.6	130.3±32.5	43.1±18.3

**(b) 3BC315 IgG**							
	***k***_***on1***_ **(ms**^−1^**)**	***K***_***off1***_ **(s**^−1^**)**	***k***_***on2***_ **(RUs**^−1^**)**	***k***_***off2***_ **(s**^−1^**)**	***K***_***d1***_ **(nM)**	***K***_***d2***_ **(RU)**	***S***_***m***_
293T (*n*=4)	4.9 × 10^3^±1.9 × 10^2^	<10^**−**5^	3.4 × 10^**−**4^±1.4 × 10^−5^	8.4 × 10^**−**3^±1.1 × 10^−3^	<10	24±2.2	1.1±0.04

**3BC315 Fab**				
	***k***_***on***_ **(ms**^−1^**)**	***K***_***off***_ **(s**^−1^**)**	***K***_***d***_ **(nM)**	***S***_***m***_
293T (*n*=3)	6.2 × 10^3^±4.0 × 10^2^	<10^**−**5^	<10	1.1±0.013
293F (*n*=2)	9.7 × 10^3^±1.1 × 10^3^	<10^**−**5^	<10	1.3±0.041
293S (*n*=2)	1.3 × 10^4^±1.1 × 10^3^	5.8 × 10^**−**5^±2.1 × 10^**−**5^	4.7±2.0	1.5±0.042
293S EndoH (*n*=2)	8.5 × 10^4^±4.6 × 10^3^	5.9 × 10^**−**4^±1.2 × 10^**−**4^	7.1±1.8	1.9±0.047

(a) Affinity and stoichiometry (N) of 3BC315 binding to various SOSIP constructs. All ITC experiments were done a minimum of two times; BG505 SOSIP.664_293S_ (n=2), BG505 SOSIP.664_293S_ deglycosylated (*n*=2), SOSIP.664_293F_ (*n*=3), BG505 SOSIP.650_293F_ (*n*=3). Based on previous ITC-binding observations where one PG9 Fab binding to the BG505 trimer results in *N*=0.6–0.8 (refs 13, 21), 2 PGT151 Fabs binding to the trimer gives *N*=1.3 (ref. 18) and 3 Fabs binding per trimer, such as PGT121 and 2G12, results in *N*=2.3–2.4 (ref. 21), the *N* value ranges shown here of 0.76–1.71 correlate with the binding stoichiometry of 1–2 Fabs per trimer observed by EM. The range indicates differences in stoichiometry for less processed (high mannose) and deglycosylated 293S trimers (upper *N* value range indicating more trimers have 2 Fabs bound), compared with more processed (complex and high-mannose glycans) 293F trimers (lower *N* values with less Fabs bound). Error values are indicated as s.d. (b) SPR binding measurements of 3BC315 IgG (top) and Fab (bottom) to various BG505 SOSIP.664 trimers. The dissociation was too slow in some of the binding curves, resulting in off-rates near the limit of detectability. Therefore, only the upper limit of the *k*_*off*_ and consequently K_*d*_values are given. The upper limits for the K_*d*_ estimated by SPR are significantly lower than the K_*d*_ values measured by ITC. Such differences have been observed for other HIV-1 antibodies, and we have previously suggested possible explanations^36^. Notably, the binding stoichiometry (S_m_) is higher when the glycans are all oligomannose (293S), and increase on deglycosylation by EndoH as observed in ITC. The corresponding SPR curves can be found in Supplementary Fig. 6. Errors are measured as s.e.m.

**Table 2:** Analysis of antibody-induced trimer decay by EM.

	**3BC176 (%)**	**3BC315 (%)**	**PGV04 (%)**
*JR-FL SOSIP.664*			
1 h	74.4	5.00	0
6 h	79.0	83.8	0
18 h	88.8	91.7	0
			
*BG505 SOSIP.664*			
1 h	5.1	0	0
6 h	64.1	82.5	0
18 h	77.7	94.9	0

A subpopulation of reference-free 2D class averages generated from negative-stain EM data after complexing JR-FL or BG505 SOSIP.664_293F_ trimers with 3BC176 or 3BC315 Fab illustrates the trimer decay. The percentage of values, which indicate the fraction of Fab-bound particles that are not in the trimeric form, increase over time for 3BC176 and 3BC315 but not PGV04.

**Table 3:** Pre-incubation neutralization assay using 3BC176 and 3BC315.

**JR-FL**						
**Pre-incubation (h)**	**IC**_**50**_ **(μg ml**^−1^**)**	**Fold change**	**IC**_**50**_ **(μg ml**^−1^**)**	**Fold change**	**IC**_**50**_ **(μg ml**^−1^**)**	**Fold change**
	**3BC176**		**3BC315**		**35O22**	
1	0.30		5.84		0.0023	
3	0.16	1.8	3.69	1.6	0.0028	0.8
6	0.11	2.7	2.34	2.5	0.0012	2.0
22	0.03	**8.7**	0.83	**7.0**	0.0015	1.5
	**sCD4**		**PGT145**		**2G12**	
1	0.61		0.0064		0.12	
3	0.33	1.8	0.0048	1.3	0.11	1.1
6	0.28	2.2	0.0041	1.5	0.13	1.0
22	0.13	**4.9**	0.0019	**3.3**	0.09	1.4
	**D5**		**T-20**			
1	1,045		0.019			
3	722	1.4	0.022	0.9		
6	1,290	0.8	0.017	1.1		
22	686	1.5	0.016	1.2		
						
**BG505**						
	**3BC176**		**3BC315**		**35O22**	
1	1.93		1.62		0.0022	
3	1.04	1.9	1.01	1.6	0.0011	2.1
6	0.82	2.4	0.71	2.3	0.0014	1.6
22	0.33	**5.8**	0.41	**4.0**	0.0011	2.0
	**sCD4**		**PGT145**		**2G12**	
1	3.05		0.0096		0.092	
3	2.74	1.1	0.0085	1.1	0.099	0.9
6	2.42	1.3	0.0099	1.0	0.101	0.9
22	1.19	2.6	0.0069	1.4	0.064	1.4
	**D5**		**T-20**			
1	125		1.17			
3	110	1.1	1.03	1.1		
6	161	0.8	0.95	1.2		
22	130	1.0	1.14	1.0		

JR-FL or BG505 virus was incubated with antibody for various times, before adding to TZM-bl cells to test for infectivity. IC_50_ values of 3BC315 and 3BC176 decrease as the antibody is pre-incubated with the viruses for longer times, indicating that these antibodies neutralize by inactivating Env over time. Fold change (decrease) in IC_50_ is calculated relative to the standard 1h incubation time. Incubations resulting in a >3-fold change are shown in boldface.
