# Supplementary material for: Antibodies to a conformational epitope on gp41 neutralize HIV-1 by destabilizing the Env spike
Source: Nat Commun. 2015 Sep 25;6:8167. doi: 10.1038/ncomms9167 (PMC4586043; doi:10.1038/ncomms9167)
Supplement: Supplementary Information — Supplementary Figures 1-7, Supplementary Tables 1-3 and Supplementary References [file ncomms9167-s1.pdf]

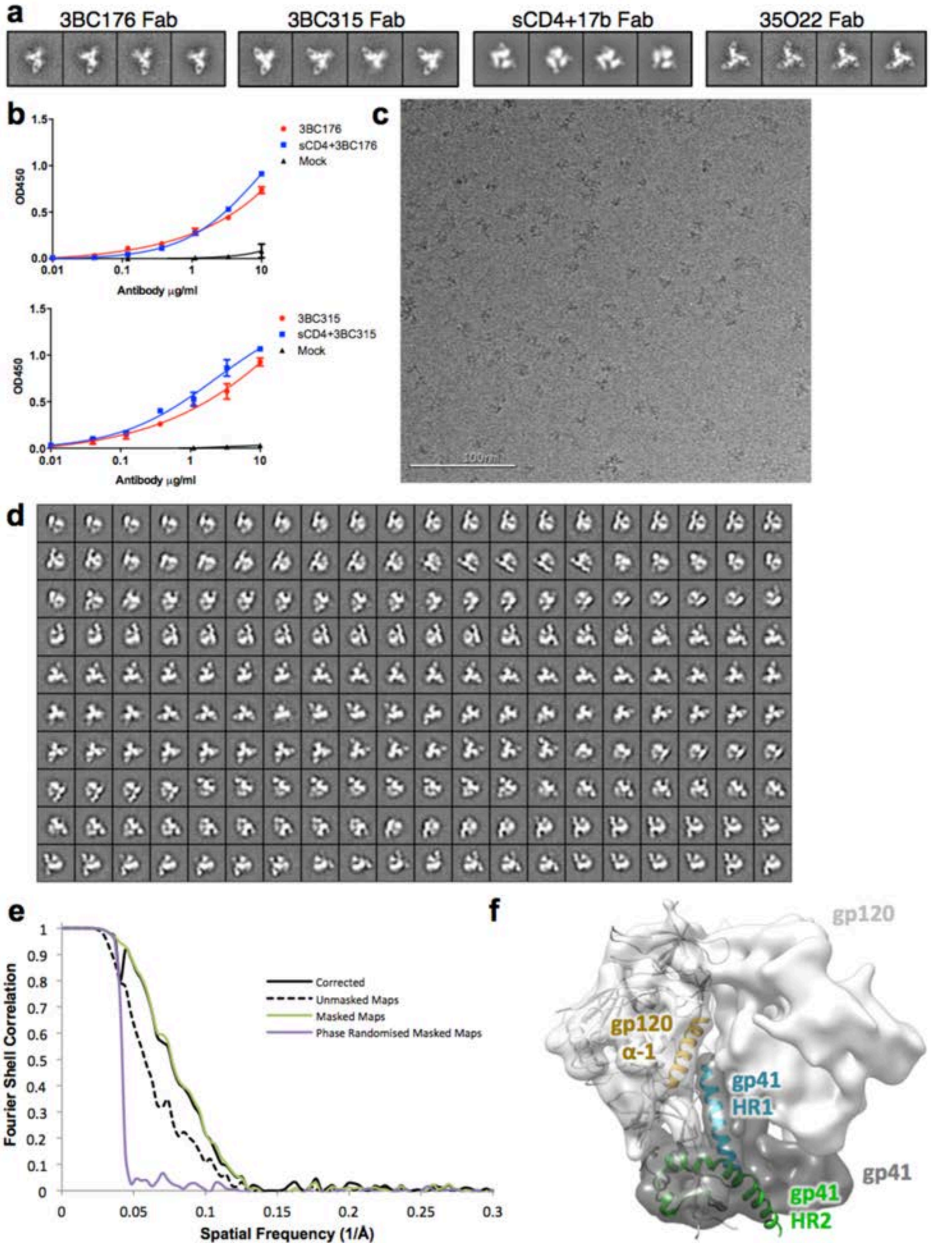


**Supplementary Figure 1. Validation that 3BC176/3BC315 binds at the gp120-gp41 interface and not at the CD4i site. (a)** Reference-free 2D class averages of 3BC176 Fab, 3BC315 Fab, sCD4 and 17b Fab, and 35O22 Fab bound to the BG505 SOSIP.664$_{293F}$ trimer. The class averages of 3BC176 and 3BC315 are more similar to 35O22 Fab, which binds the gp120-gp41 interface, than to co-receptor binding site (CD4i) antibody, 17b Fab. **(b)** ELISA curves show similar binding of 3BC315 or 3BC176 to BG505 SOSIP.664$_{293T}$ in the presence or absence of sCD4. Classic CD4i antibodies have enhanced binding upon CD4 binding. **(c)** A cryoEM micrograph at 2.12 µM underfocus. A scale bar corresponding to 100 nm is shown at the bottom left. **(d)** Reference-free 2D class averages generated from the cryoEM data. **(e)** Gold-standard Fourier-shell correlation (FSC) curves of the processed data. The FSC curve from the unmasked maps shown as the dotted line is generated from the unprocessed two half reconstructions from the refinement. The FSC calculated from phase-randomized two halves after applying a model-shaped mask with a Gaussian fall-off is shown in green. The masking-effect on the FSC is measured by analyzing the resolution difference between the phase-randomized and non-randomized FSC curves, as shown in purple. The final FSC curve corrected is shown in black. At an FSC cut off of 0.143, the final resolution is 9.3 Å. **(f)** Upon docking the BG505 SOSIP.664 gp140 structure (PDB ID: 4TVP), the large helical structures fit nicely into the density in the EM density, including helix α-1 in gp120 (yellow), gp41 HR1 helix (blue), and gp41 HR2 helix (green). The map has been segmented to remove the 3BC315 Fabs for clarity, and to distinguish between gp120 density (white) and gp41 density (gray).

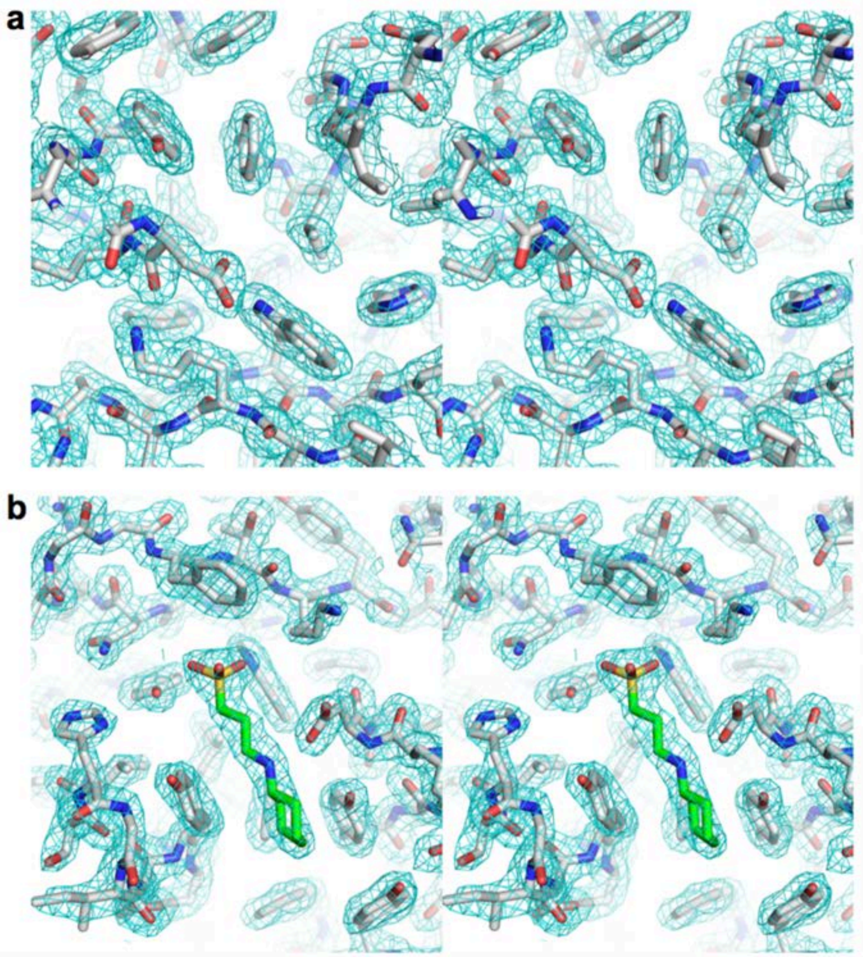


**Supplementary Figure 2. Stereo images of the electron density maps of the 3BC176 and 3BC315 Fabs.** The 2Fo-Fc map at σ=1.00 is shown for **(a)** 3BC176 and **(b)** 3BC315.

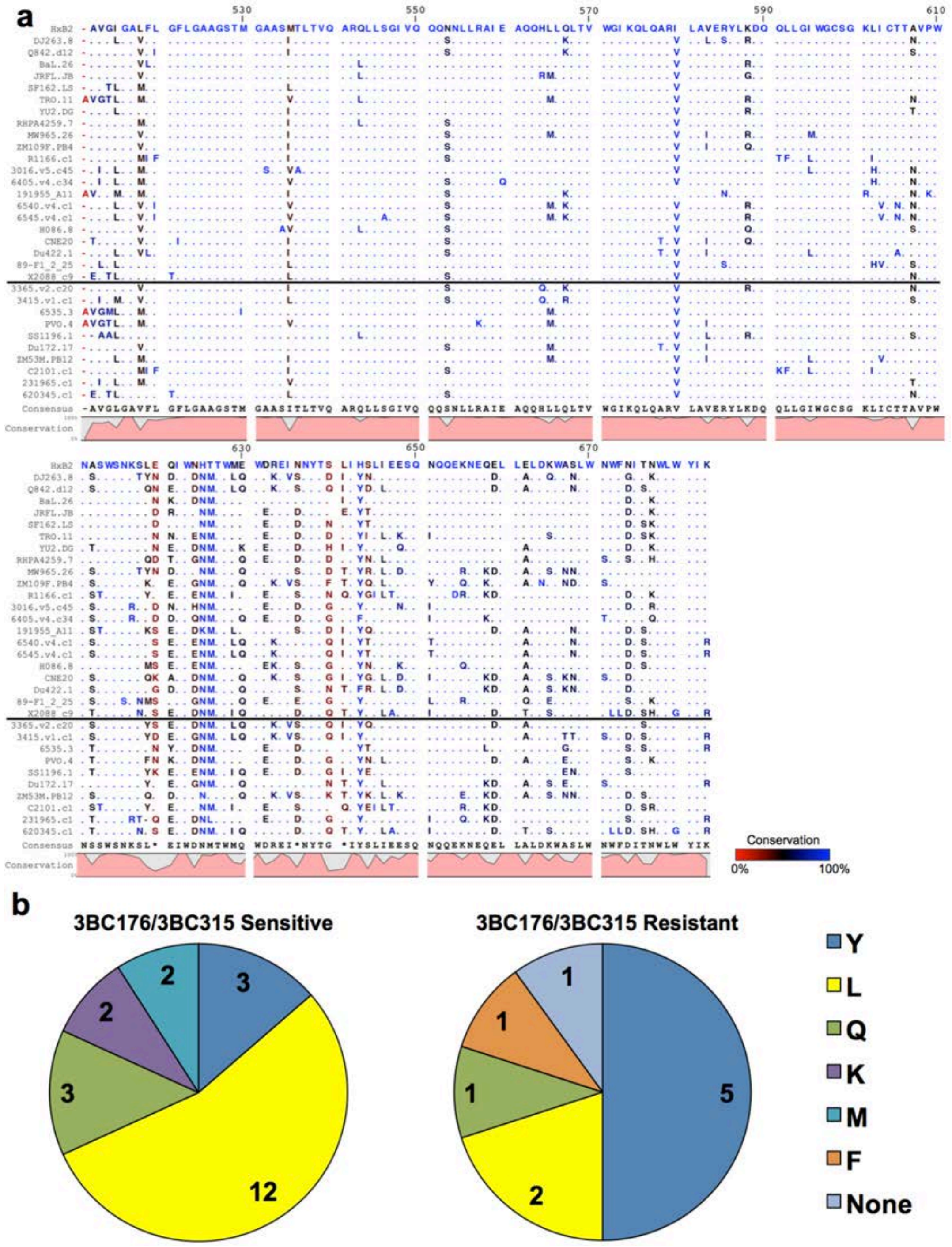


**Supplementary Figure 3. Sequence comparison between Envs that are sensitive and resistant to 3BC315 and 3BC176. (a)** Peptide sequence alignment of the extracellular gp41 region of the viruses in the neutralization panel by Klein *et al.*[1]. The sequences have been aligned relative to HxB2 as a reference, and residues that are identical to the HxB2 sequence at given positions are shown as dots. Isolates that are sensitive to (upper) or resistant to (lower) 3BC176/3BC315 are separated by a black line. Degree of sequence conservation is shown by residue coloring, as indicated by the key at the bottom right. **(b)** Sequence variability at position 619 of gp41 in 3BC176/3BC315 sensitive (left) or resistant (right) viruses. The total number of sensitive and resistant isolates shown here is 22 and 10, respectively.

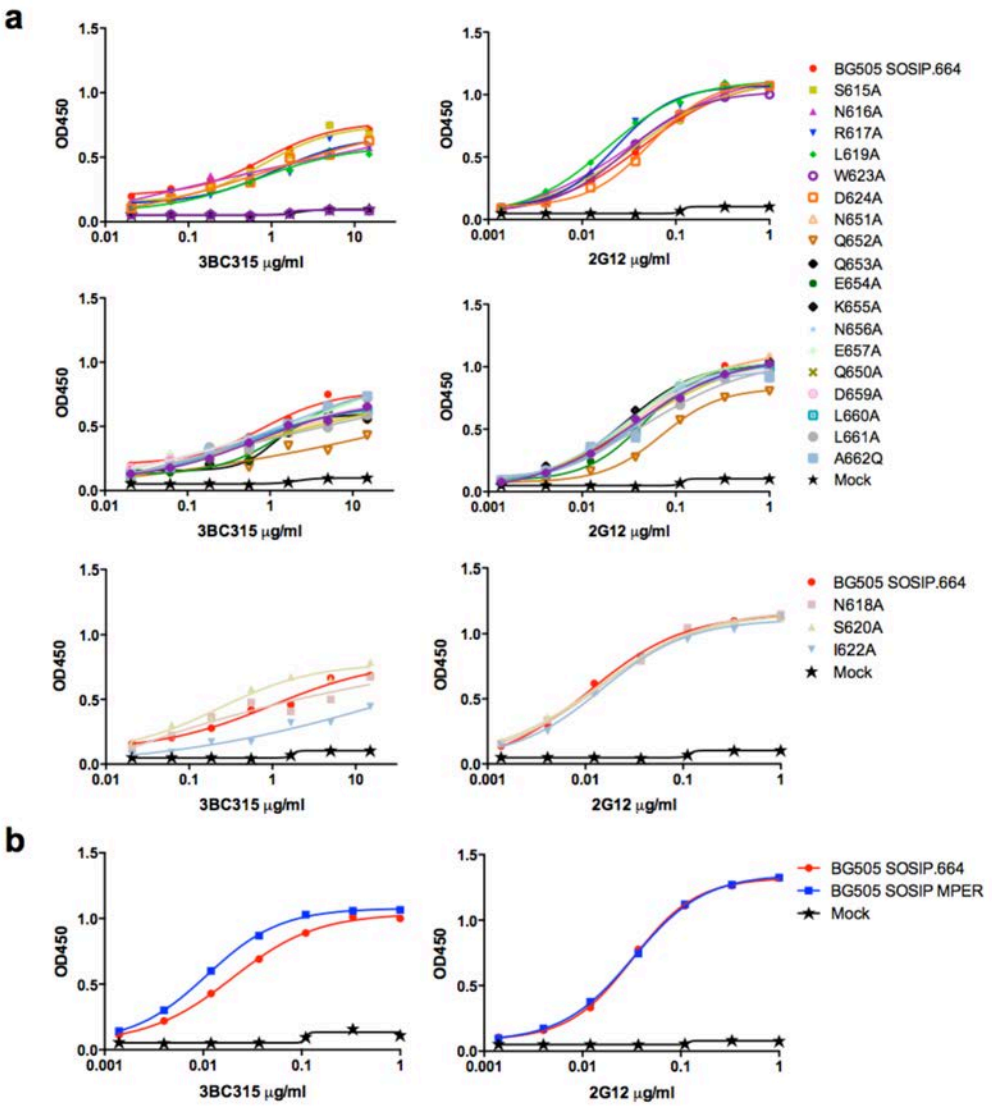


**Supplementary Figure 4. Effect of gp41 modifications in 3BC315 binding by ELISA (a)** ELISA curves showing 3BC315 binding to various point mutations in the HR2 region in BG505 SOSIP.664$_{293T}$. Most mutations have small (I622A, Q652A) or no effects on binding except for the W623A mutation. 2G12 is used as a control, as it is a gp120-binding antibody. **(b)** 3BC315 binding to BG505 SOSIP$_{293F}$ with and without the MPER. Only a slight increase in 3BC315 binding is seen upon adding back the MPER. 2G12 is used as a control antibody.

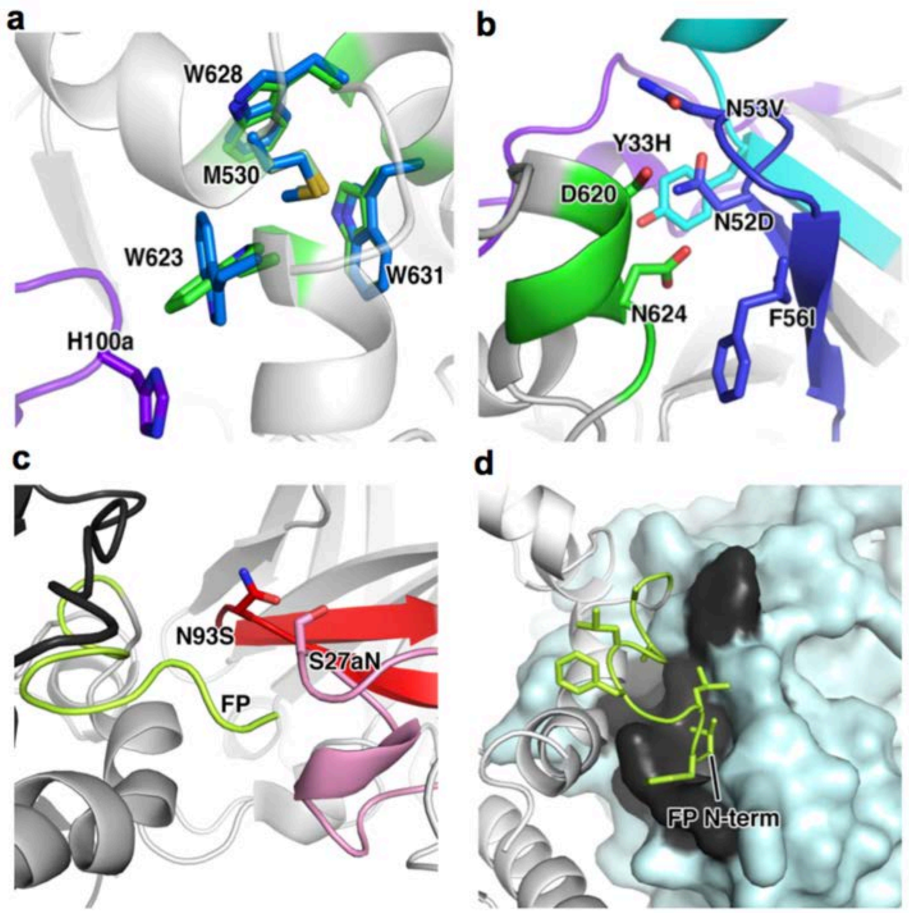


**Supplementary Figure 5. Different modes of epitope recognition by 3BC176 and 3BC315. (a)** H100a in CDRH3 (purple), a residue conserved in both 3BC315 and 3BC176, may interact with W623 to disrupt the tryptophan clasp. The position of the tryptophan clasp residues in the 4TVP crystal structure (blue) superimposed onto our Rosetta refined model (green). In our model, only the W623 rotamer is different and the W623 indole is no longer perpendicular to M530. Although direct interactions are not seen in our model, H100a likely causes this displacement and may destabilize the trimer. **(b)** Potential contacts between HR2 (green) and Fab HC residues that differ in 3BC176 and 3BC315 are mapped onto the structure of 3BC315 Fab. The corresponding amino acid in 3BC176 is shown second. Residue 620 is a serine in BG505, and aspartic acid in JR-FL **(c)** Potential contact residues for the LC, which likely interacts with the FP (lime green). **(d)** Hydrophobic residues (black) in 3BC315 (light blue surface) near the FP (lime green) form a shallow pocket that may accommodate the FP. The residues in this region are conserved between 3BC176 and 3BC315. In the model shown, six residues at the N-terminus of the FP are missing. The CDR loops are colored as follows: CDRH1: cyan, CDRH2: blue, CDRH3: purple, CDRL1: pink, CDRL3: red.

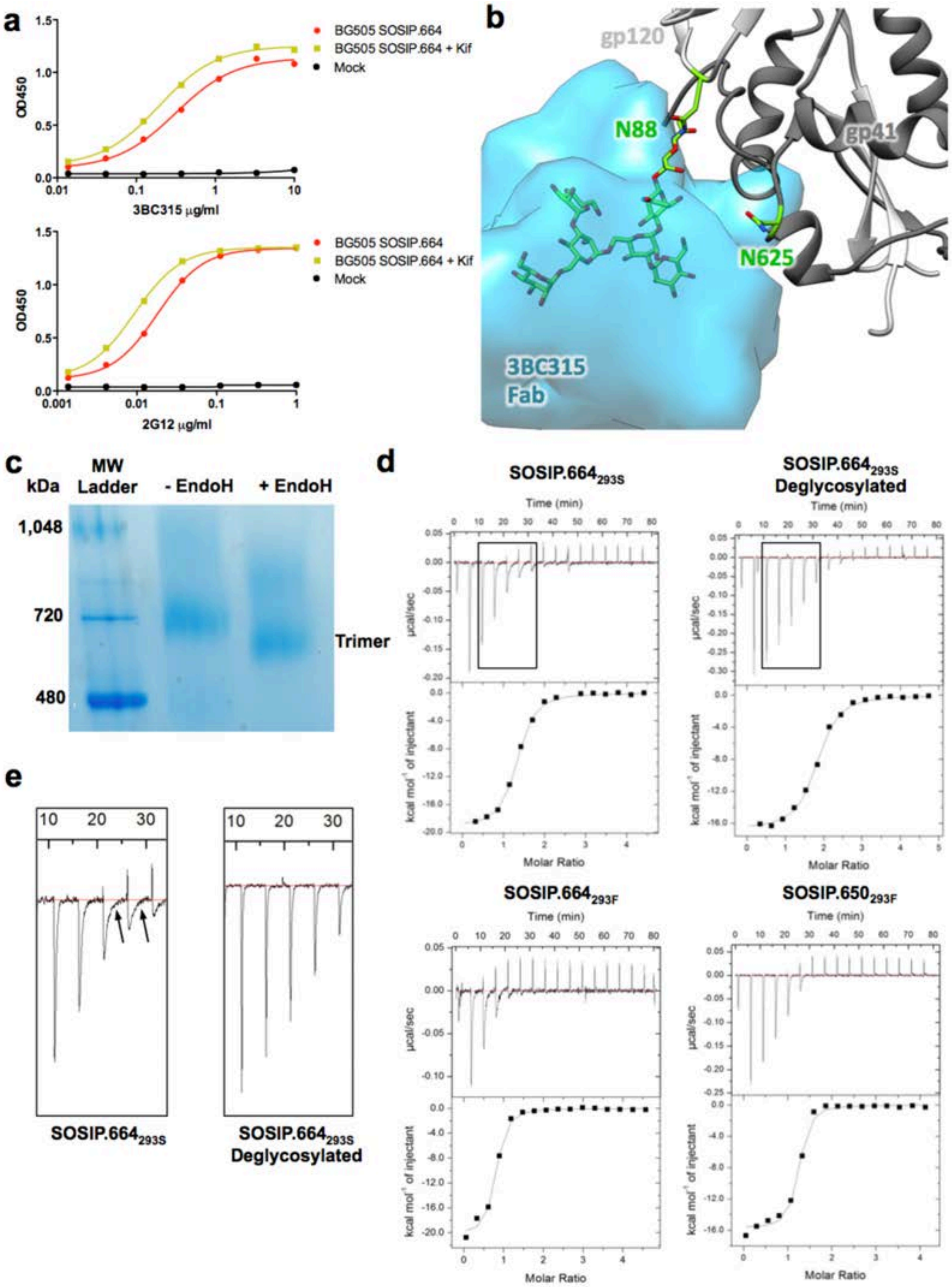


**Supplementary Figure 6. Effect of glycans on 3BC315 Fab binding. (a)** 3BC315 binding to BG505 SOSIP.664$_{293T}$ expressed in the presence of kifunensine (Kif) slightly increases the binding of 3BC315 to the trimer by ELISA and shows that 3BC315 binding is not dependent on complex glycans. The control antibody 2G12 is known to bind the high-mannose patch. **(b)** Docking of the BG505 SOSIP.664 trimer structure (PDB ID: 4TVP) into the EM map shows that the N88 glycan would clash with 3BC315 Fab, unless the glycan orientation is changed. A glycan on N625 could also sterically hinder 3BC315 binding, but no glycan on N625 is observed in the X-ray structure and hence is not modeled. **(c)** Native gel showing the BG505 SOSIP.664$_{293S}$ trimer before and after EndoH treatment. **(d)** Exemplar ITC curves of 3BC315 binding to various SOSIP constructs, where the Fab is titrated into the BG505 SOSIP trimer. Binding affinities are summarized in Table 1a. A closer look at ITC heat recovery signals (boxed) from the ITC curves is shown in **(e)**, where the broad heat signals (arrows) in the glycosylated sample compared to the deglycosylated material indicates that 3BC315 binds the fully glycosylated trimer with a slower on-rate than to the deglycosylated trimer.

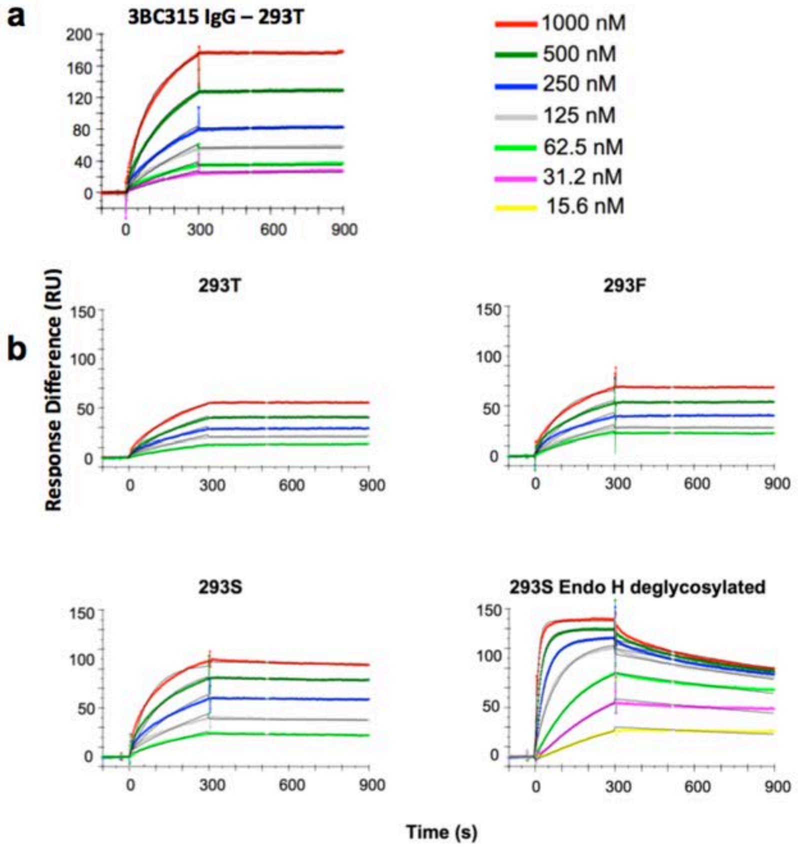


**Supplementary Figure 7. SPR analysis of the rate of 3BC315 binding. (a)** BG505 SOSIP.664 trimers produced in 293T cells were immobilized via a His-tag. 3BC315 IgG were bound to the trimer at varying titration concentrations as indicated. **(b)** The same experimental setup was carried out to test 3BC315 Fab binding to trimers produced in 293T cells, 293F cells, 293S cells, or 293S cell produced trimers deglycosylated by treatment with EndoH. The binding curves to the deglycosylated trimer have a distinctively steep slope, suggesting a rapid on-rate. The kinetic values calculated from these curves are listed in Table 1b. Different colored curves correspond to different Fab titrations as indicated by the key in the top right. The fitted curves obtained by a Langmuir model are shown as thin black lines.

| | 3BC315 Fab | 3BC176 Fab |
|---|---|---|
| **Data collection** | | |
| Space group | $P2_12_12_1$ | $P2_1$ |
| Cell dimensions | | |
| *a*, *b*, *c* (Å) | 48.08, 49.52, 159.03 | 41.7, 60.9, 87.2 |
| a, b, g (°) | 90, 90, 90 | 90, 95.4, 90 |
| Resolution (Å) | 50.00-1.95 (1.98-1.95) | 50.00-1.89 (1.92-1.89) |
| $R_{sym}$ | 5.3 (11.5) | 9.6 (45.1) |
| $I$ / σ$I$ | 61.2 (31.2) | 16.7 (2.6) |
| Completeness (%) | 96.8 (92.8) | 99.7 (98.5) |
| Redundancy | 6.2 (5.9) | 3.7 (3.5) |
| | | |
| **Refinement** | | |
| Resolution (Å) | 47.28-1.95 | 49.91-1.89 |
| No. reflections | 27,626 | 35,026 |
| $R_{work}$ / $R_{free}$ | 15.6/20.7 | 17.8/22.4 |
| No. atoms | | |
| Protein | 3,318 | 3,292 |
| Solvent | 353 | 306 |
| *B*-factors | | |
| Protein | 20.2 | 23.2 |
| Solvent | 26.2 | 27.8 |
| R.m.s. deviations | | |
| Bond lengths (Å) | 0.007 | 0.007 |
| Bond angles (°) | 1.18 | 1.17 |

**Supplementary Table 1. Data collection and refinement statistics for 3BC315 and 3BC176 Fab crystal structures.** Values in parentheses are for highest-resolution shell. Each structure was solved by molecular replacement with data from a single crystal.

| (a) | JR-FL/JR2* | |
|---|---|---|
| Mutation | Fold IC$_{50}$ Increase | |
| gp120 | 3BC176 | 3BC315 |
| N88A* | 0.03 | 0.03 |
| E492A | 0.45 | 2.7 |
| P493A | 0.43 | 4.2 |
| L494A | 0.03 | 0.92 |
| G495A | 0.35 | 2.1 |
| V496A | *ni*[‡] | *ni* |
| A497G | 0.16 | 1.9 |
| P498A | *ni* | *ni* |
| T499A | 0.02 | *nd*[†] |
| T499S | 0.30 | *nd* |
| K502L | 0.90 | *nd* |
| gp41 | 3BC176 | 3BC315 |
| Q590A | 0.27 | 2.3 |
| Q591A | *ni* | *ni* |
| L592A | 0.42 | 1.8 |
| L593A | 0.09 | 1.5 |
| G594A | 0.37 | 2.3 |
| I595A | 0.05 | 0.22 |
| W596A | 0.1 | 1.0 |
| G597A | 0.06 | 0.22 |
| S599R | 0.25 | *nd* |
| L602H | 0.12 | *nd* |
| I603V | 0.09 | *nd* |
| T605Y | 0.40 | *nd* |
| N611A | 0.18 | 0.93 |
| N611D | 2.8 | 0.88 |
| A612G | 0.28 | 1.8 |
| S613A | 0.55 | 1.4 |
| W614A | *ni* | *ni* |
| S615A | 0.74 | 3.2 |
| N616A | 0.86 | 2.5 |
| N616S | 6.5 | 2.0 |
| K617A | 1.1 | 3.8 |
| S618A | 0.83 | 3.5 |
| L619A | 0.65 | 4.2 |
| L619Q | 1.4 | *nd* |

| | JR-FL/JR2* | |
|---|---|---|
| Mutation | Fold IC$_{50}$ Increase | |
| gp41 | 3BC176 | 3BC315 |
| L619Y | 15.1 | >17.5 |
| D620A | 0.31 | 4.0 |
| R621A | 0.97 | 3.1 |
| W623L | 52.4 | 8.3 |
| N624A | 0.3 | 2.3 |
| N625Q | 3.5 | 7.6 |
| M629A | 0.73 | 1.4 |
| E630A | 0.56 | 1.2 |
| W631A | *ni* | *ni* |
| E632A | 1.2 | 1.0 |
| R633A | 1.1 | 0.92 |
| E634A | 0.53 | 1.1 |
| I635A | *ni* | *ni* |
| D636A | 1.0 | 0.98 |
| N637K | 5.5 | 2.6 |
| E641A* | 4.5 | 4.0 |
| I642A* | *ni* | *ni* |
| Y643A* | 0.87 | 2.4 |
| T644A* | 5.8 | 5.0 |
| L645A* | 8.7 | 2.5 |
| I646A* | 0.21 | 0.51 |
| E647A* | 1.0 | 1.8 |
| E648A* | 9.9 | 5.7 |
| E648K | 44.1 | 2.5 |
| E648Q | 40.6 | 2.3 |
| S649A* | *ni* | *ni* |
| Q650A* | 0.89 | 2.5 |
| N651A* | *ni* | *ni* |
| Q652A* | 4.7 | 3.6 |
| Q653A* | 2.0 | 3.4 |
| E654A* | 2.0 | 3.4 |
| K655A* | 1.8 | 3.5 |
| N656A* | 13.1 | 3.8 |
| A667E* | 1.5 | 1.0 |
| F673A* | 0.15 | *nd* |
| W678A* | 0.32 | *nd* |

| (b) | BG505 | |
|---|---|---|
| Mutation | Fold IC$_{50}$ Increase | |
| gp41 | 3BC176 | 3BC315 |
| L619Y | >77.6 | 54.9 |
| I641A | 0.74 | 1.3 |
| I642A | *ni* | *ni* |
| Y643A | 1.5 | 1.2 |
| G644A | *ni* | *ni* |
| L645A | 0.72 | 1.3 |
| L646A | *ni* | *ni* |
| E647A | 0.26 | 1.0 |
| E648A | 1.7 | 1.1 |
| S649A | 1.0 | 1.4 |
| Q650A | <0.04 | 0.24 |
| N651A | <0.09 | 0.52 |
| Q652A | 0.7 | 0.93 |
| Q653A | <0.1 | 0.48 |
| E654A | <0.2 | 0.89 |
| K655A | <0.02 | 0.01 |
| N656A | 1.0 | 1.6 |

**Supplementary Table 2. Change in neutralization sensitivity of various mutants** of **(a)** JR-FL/JR2 and **(b)** BG505 pseudotyped virus (IC$_{50}$ wild-type/IC$_{50}$ mutant) against 3BC176 and 3BC315 in a TZM-bl neutralization assay. [‡] *ni:* not infectious. [†] *nd:* not determined. *These mutants were tested on a JR2 background; all other mutations were tested on JR-FL. Color scheme: blue = >4-fold decrease in IC$_{50}$, yellow = 4-10-fold increase in IC$_{50}$, red = >10-fold increase in IC$_{50}$.

| Region of gp41 | ID | AA Sequence | EC50 (μg/ml) | |
|---|---|---|---|---|
| | | | 3BC176 | 3BC315 |
| Fusion Peptide | FP | AVGIGALFLGFLGAAGSTMGARSKKK | >10 | >10 |
| NHR | 9108 | LSGIVQQQSNLLRAI | >10 | >10 |
| | 9111 | RAIEAQQHLLQLTVW | >10 | >10 |
| | 265 | [Ac-IEAQQHLLQLTVWGIKQLQARIEA-IZ-NH2]3 | >10 | >10 |
| Disulfide Loop | PID | LLGIWGCSQKLICTTAVPWKKK | >10 | >10 |
| CHR | 9126 | KSQDEIWDNMTWMEW | >10 | >10 |
| | 9127 | EIWDNMTWMEWEREI | >10 | >10 |
| | 9128 | NMTWMEWEREINNYT | >10 | >10 |
| | 9129 | MEWEREINNYTDIIY | >10 | >10 |
| | 9130 | REINNYTDIIYSLIE | >10 | >10 |
| | 9131 | NYTDIIYSLIEESQN | >10 | >10 |
| | 9132 | IIYSLIEESQNQQEK | >10 | >10 |
| | 9133 | LIEESQNQQEKNEQE | >10 | >10 |
| | 9134 | SQNQQEKNEQELLAL | >10 | >10 |
| | C34 | WMEWDREINNYTSLIHSLIEESQNQQEKNEQELL | >10 | >10 |
| | T20 | YTSLIHSLIEESQNQQEKNEQELLELDKWASLWNWF | >10 | >10 |
| MPER | C1 | QIQQEKNMYELLALDKWASLWNWFDITKWLWYIKYGVYIVK | >10 | >10 |
| | PDT-081 | EKNEQELLELDKWASLWNWFDITNWLWYIKKKK | >10 | >10 |
| | 179-4 | LLELDKWASLWNWFDITNWLWYIKKKK | >10 | >10 |
| **Protein** | | | **3BC176** | **3BC315** |
| 5-Helix | | | >10 | >10 |
| JRFL gp41-MBP fusion (M41xt) | | | >10 | >10 |
| JRFL gp41 I559P (M41xt I559P) | | | >10 | >10 |
| ADA gp140 | | | >50 | >50 |
| ADA gp140 I559P | | | 1.7 | 0.05 |
| ADA gp140 + sCD4 | | | >10 | >10 |
| JRFL gp120 | | | >10 | >10 |
| JRFL gp120 + sCD4 | | | >10 | >10 |
| Ovalbumin | | | >10 | >10 |

**Supplementary Table 3. 3BC176 and 3BC315 IgG binding to various gp41 polypeptides by ELISA.**

**Supplementary References**


1. Klein, F. et al. Broad neutralization by a combination of antibodies recognizing the CD4 binding site and a new conformational epitope on the HIV-1 envelope protein. *J Exp Med* **209**, 1469-1479 (2012).
